# A WSN Layer-Cluster Key Management Scheme Based on Quadratic Polynomial and Lagrange Interpolation Polynomial

**DOI:** 10.3390/s20164388

**Published:** 2020-08-06

**Authors:** Xiaogang Wang, Zhongfan Yang, Zhiqiang Feng, Jun Zhao

**Affiliations:** 1 School of Automation & Information Engineering, Sichuan University of Science & Engineering, Yibin 644000, China; yang56535@163.com (Z.Y.); jonathan_fzq@163.com (Z.F.); Zhaojun@suse.edu.cn (J.Z.); 2 Artificial Intelligence Key Laboratory of Sichuan Province, Yibin 644000, China

**Keywords:** layer-cluster key, quadratic polynomial, Lagrange interpolation polynomial, key management, wireless sensor network

## Abstract

Since current key management schemes are mainly designed for static and planar networks, they are not very suitable for the layer-cluster wireless sensor networks (WSNs), a WSN layer-cluster key management scheme based on quadratic polynomial and Lagrange interpolation polynomial is proposed, in which the main idea of this scheme along the research line of broadcast identity authentication, session key, group key, network key and personal key. Specifically, authentication key can be established on the basis of Fourier series for identity authentication; session key is established by a multiple asymmetric quadratic polynomial, in which session key information is encrypted by the authentication key to ensure the security of intermediate interactive information; based on the former two keys, group key is established on the basis of Lagrange interpolation polynomial, in which the nodes of the cluster are not directly involved; the generation and management of network key is similar to the group key, in which the establishment idea is to regard the BS and all cluster heads as a group; the generation and management of personal key is also similar to the group key, the difference is that the personal key can be obtained by cluster nodes through getting the Lagrange interpolation polynomial coefficients based on their own random key information. It is analyzed that the proposed layer-cluster key management scheme can guarantee the identity of network nodes firstly through forward authentication and reverse authentication, and session key, group key and network key will guarantee the independence of the keys’ management and avoids the problem of single point failure compared with LEAP protocol, and personal key will guarantee the privacy of network.

## 1. Introduction

The development of modern network technology has proved a fact that a network without enough security cannot guarantee the future of a network [1,2]. Wireless Sensor Networks (WSNs) as a new network technology originated from the military field, require more attention to security [3,4]. Due to the great difference between WSNs and traditional networks, WSN security problems have some new characteristics: (1) because of the characteristics of self-organization, intermittent connection, wireless communication and resource limitation, it is difficult for WSN to fully guarantee the network security [5]; (2) WSN is vulnerable to be threats from internal, external and malicious attacks [6]; (3) the information and resources of WSN can be modified, eavesdropped, deleted, lost or disclosed, and the service may be blocked, or even the environment is not safe and vulnerable [7]. So, the key research of WSN security is to provide a service including self-protection, reliability, confidentiality, authenticity, and integrity service.

Since the characteristics of WSN determine that the security problems of WSN are much different from the traditional network [4,7], and the unreliable wireless communication channel makes WSN security execution more difficult. Even in some military special environments, WSN nodes are required to have the ability to detect and identify untrusted nodes and intruders and can resist various types of attacks for maintaining the security and integrity of the network. All these problems require WSN to have a higher and stronger security mechanism to overcome the weakness of WSN in security and ensure the application of WSN in various fields.

For WSN security, the actual situation is that the open wireless channel needs an encryption system, and the wireless sensor nodes constrained by resources need a lightweight and efficient security scheme, and the characteristic of uncontrolled operation of WSN needs a security strategy with high security flexibility [8]. At present, almost all encryption technologies rely on keys, but the leakage of the keys will directly lead to the leakage of the plaintexts. Therefore, key management is the key part of guaranteeing the wireless communication, and how to configure and manage keys effectively and safely has become one of the important parts of WSN security research.

At present, the research on security technologies of WSN involves cryptography, key management, data security fusion, security routing, intrusion detection, identity authentication, trust model and other special security issues [4,7], where the key management scheme is the most critical issue and also the basis of other security mechanisms such as secure routing, secure location, secure data fusion, etc., but the key management technology is also the most difficult and weak part of WSN security management [9]. It is shown in historical examples that the attack cost of key management is much less than the decoding algorithm. Therefore, in WSN security research, it is very important to attach great importance to the key management and introduce the key management schemes for effective control, which can increase the security and anti-attack of the network [10,11,12].

### 1.1. Identity Authentication

For key management research, researchers rarely classify the identity authentication as a key management technology. It is known that the broadcast identity authentication is the first secure task when a WSN begins to run, which can guarantee the sources of network information and conduct a periodic confirmation in subsequent work. In fact, the classic algorithms such as hash chain and digital signature authentication are essentially a process of key management [13,14,15]. Therefore, this paper proposes a layer-cluster key management scheme which takes the broadcast identity authentication as the first work of key management, the broadcast identity authentication work runs through the whole process of key management. For example, the network initialization requires the broadcast identity authentication, and identity authentication is also required when the network is periodically updated or attacked abnormally. In addition, broadcast identity authentication is the first secure barrier of WSN network, in which the broadcast authentication key generated at the first step can be used to encrypt the later key information and participate the generation of other keys.

In WSN, in order to save the network bandwidth and the communication time, base station (BS) or cluster heads usually send commands or make updating by means of broadcast. Since the broadcast communication plays a very important role in WSN and its security is directly related to the security of the whole network, it must be able to authenticate the source, accuracy and integrity of the broadcast packet when a node receives a broadcast packet, which also known as the broadcast authentication.

Broadcast authentication includes entity authentication and message source authentication. Entity authentication is a process in which one party confirms the identity of the other party according to a certain protocol. Message source authentication is mainly to confirm the legal identity of the information source and ensure the integrity of the information, which can prevent illegal nodes from sending, forgery and tampering with the information. These two parts of broadcast authentication can be realized by encrypting and decrypting the message authentication code (MAC).

Because of the limited energy, computing power, storage capacity and mobility of WSN nodes the traditional broadcast authentication protocol cannot be applied directly, so it is urgent to design a corresponding broadcast authentication protocol according to the above characteristics. Currently, many energy-efficient broadcast protocols and algorithms have been proposed [16,17,18,19,20], and there are two main WSN broadcast authentication ways: one is signature authentication [15], but the disadvantage of this way is that it uses the public key cryptography which is expensive and hard to be applied in WSN; the other way is based on the message authentication code (MAC), such as one-way hash chain method and the µTESLA protocol proposed by Perrig according to the SPIN security model [5], in which the µTESLA protocol can realize asymmetric authentication based on the delay authentication, but the delay increases gradually with the time change.

### 1.2. Session Key

Session key is an encryption and decryption key generated for the secure communication between the neighbor nodes of the network or every two members of the group. Session key is generally symmetric, which means that the encryption and decryption keys are same and is known as unicast key. Generally, a secure communication channel can be established based on the session key after finishing the identity authentication. The management of session key includes keys generation, distribution, updating and revocation.

Session key is the commonly understood form of key management, and its establishment and research are generally based on the distributed network structure. At present, researchers have proposed a variety of WSN session key management schemes, mainly including three types:

(1) The key pre-distribution schemes based on keys pool, such as the key management scheme for distributed sensor networks proposed by Eschenauer-G1igor [21], q-composite random key pre-distribution scheme [22], pair-wise keys in distributed sensor networks [23], etc. In these schemes, each node selects several keys from the key pool randomly and only communicates with the nodes with one or more same keys. Simple application, small computing load and supporting the dynamic changes of the network are the advantages of this type. However, because the key sharing rate between nodes is low and these schemes do not support identity authentication, attackers can easily carry out various malicious attacks by using the obtained key information.
(2) The key pre-distribution schemes based on polynomial keys pool, such as the key pre-distribution in wireless sensor networks using multivariate polynomials [24], the key pre-distribution scheme based on matrix [25,26] and the key pre-distribution scheme based on configuration knowledge [27,28], etc. These schemes are generally able to resist capture attacks and have high security and good network connectivity, but they have large calculation cost and do not support identity authentication of neighbor nodes, and the network scalability is not strong to be good for the new nodes joining.
(3) Other pre-distribution key schemes, such as the grid-based key pre-distribution scheme [29], the key management scheme based on logical key tree, etc. Although these schemes have high network connectivity and small storage cost, they have poor network applicability and security.

These above session key schemes are basically based on the symmetry of key and have certain rules to follow, while it is also a breakthrough point for attackers.

### 1.3. Group Key

Since the communication mode of BS and cluster heads is usually carried out by broadcasting, a secure group key management mechanism is very suitable for WSN communication mode. Encrypting the multicast message with group key is a way to guarantee the multicast message confidentiality, in which the key used for encryption and decryption is only known by the group members and only group members can get the encrypted message.

Multicast communication has more security threats than point-to-point unicast communication, and the characteristics of the open channel make it vulnerable to be eavesdropped by attackers, while the traditional multicast security schemes are not fully applicable to WSN, so it is important to find a safe and efficient group key management scheme for wireless sensor network.

At present, some energy-efficient group key management schemes have been proposed for WSN. For example, in [30,31,32,33,34,35], some group key management schemes based on key tree are proposed for WSN, but the performance of these schemes is limited by the structure of key tree. In [30], the EBS scheme (exclusion basis systems, EBS) using combinatorial mathematics theory is proposed for group key management, and a group key management scheme based on EBS and t-degree binary polynomials is proposed in [31], but the problem that EBS is vulnerable to collusion attack is not considered in these schemes. The logical key hierarchy (LKH) scheme supports deleting multiple members at once and has the ability to prevent the deleted members from jointly negotiating to obtain the new group key [32,33,34,35], but the group controller (GC) is responsible for all the security management, which is vulnerable to form a bottleneck problem called single point failure. Based on the LKH scheme, a group key distribution scheme based on the geographic information and routing information of nodes is proposed [34], which consumes less energy to distribute and update the group key than LKH scheme, but there is also the problem of single point failure. In addition, a new hierarchical key management scheme based on node mobility is proposed on the basis of distributed binary logic key tree [36], which guarantees the stability of nodes and reduces the cost of updating the key tree when nodes leave, but it does not consider the factor such as the residual energy of nodes, which can makes some nodes dead for running out of energy.

### 1.4. Network Key

The network key is the communication key shared by BS and all network nodes, which is similar with the group key in understanding if the whole network is seemed as a group. The network key can be used for the information that all members need to know, such as the networking command. The distribution method of network key is clear that BS encrypts it by session key and sends it to each cluster head one by one firstly, and then each cluster head re-encrypts it with its own group key and broadcasts it to each group member.

Network key is established after the establishment of session key and group key, and not all networks have the requirements of network key. Since its establishment method is similar with the group key, for preventing collusion attack, it is suggested in this paper that the network key should be limited in cluster heads and the group key should still be used in group members for broadcasting.

### 1.5. Personal Key

A personal key is a key shared by a common member node and BS, which is used by the common node to send some important secret information to BS independently, such as military secrets, abnormal data, monitoring data from the coverage area. This important information is only expected to be known by BS, and the personal key and the establishment method cannot be known by the intermediate transmission node and cluster head nodes. It can be shown that the difficulty of the personal key research lies in the security of key’s distribution and updating, and if the malicious nodes obtain too much relevant information through disguising as intermediate nodes, they will work out the key information of the personal key. Therefore, it is supposed that the establishment and updating method of personal key should maintain certain independence.

Personal keys are not required for all network management cases either and are only used in some special task situations and higher security circumstances. At present, the research on personal keys is mainly based on the definition of session key, and BS is treated as a non-adjacent node. The disadvantage of this way is that the establishment method of personal key is not independent enough, and the personal key will be cracked once the session key is cracked.

### 1.6. Layer-Cluster Key

These above key management schemes are mainly designed for static and planar networks, which are not very suitable for layer-cluster wireless sensor networks. For layer-cluster schemes, network nodes are divided into several clusters, where the cluster heads are usually powerful and the keys distribution, negotiation and updating of the common sensor nodes are all charged by cluster heads. Compared with the distributed key management schemes, these layer-cluster schemes have lower requirements on computing and storage capacity of common nodes [37]. In particular, the network has good scalability and invulnerability.

Layer-cluster key research includes the key’s generation, distribution, updating, deletion, association, efficiency, and feasibility. At present, some key-cluster key schemes have been proposed [8,37,38,39]. Zhu has proposed a LEAP scheme [8], which includes four types of communication keys. Although LEAP can achieve certain security performance, it still does not solve the problem of large energy consumption of key updating and suffers from single-point failure problem. In addition, these schemes are based on the case of fixed cluster head, which can cause huge security problems once the cluster head is captured. In a word, there are many new challenges for layer-cluster key research and providing a secure and reliable WSN key management has been becoming the most important and basic content for WSN security research.

### 1.7. Motivations

The motivations of this paper can be summarized as follows:

- Since almost all existed encryption technologies rely on keys, and the leakage of the keys will directly lead to the leakage of the plaintexts, so key management is the key part of guaranteeing wireless communication security and how to configure and manage keys effectively and safely has become one of the important parts of WSN security research.
- Key management is one of the most critical issues for security, and it is the basis of other security mechanisms such as secure routing, secure location, secure data fusion, etc. Therefore, it is very important to attach great importance to the key management and introduce appropriate key management schemes for effective control.
- The current key management schemes are mainly designed for static and planar networks and easy to be trapped in the problem of single point failure, which is not very suitable for the layer-cluster wireless sensor network (WSN).

A WSN layer-cluster key management scheme based on a quadratic polynomial and a Lagrange interpolation polynomial (LCKMS-QPLIP) is proposed in this paper and the main research idea of LCKMS-QPLIP along the line of broadcasting identity authentication, session key, group key, network key and personal key, where each key establishment method of this scheme is independent, different and the encryption process is related to each other. This scheme not only can ensure the independence of each encryption process, but also can ensure the consistency of security strength.

In addition, the layer-cluster key management scheme LCKMS-QPLIP proposed in this paper should guarantee the identity of network nodes firstly through forward authentication and reverse authentication, and session keys, group keys and network keys should guarantee the security and efficiency of the network, and personal keys should guarantee the privacy of the network. These five keys should complement each other, which will only should ensure the independence of the keys’ management and avoid the problem of single point failure, but also enable WSN to provide an efficient key management scheme in a reasonable network structure.

### 1.8. Main Contributions

The main contributions of this paper can be summarized as follows:

- Broadcast authentication. The broadcast authentication protocol based on Fourier series for WSN is used for identity authentication. The authentication key is established by the initial sharing function f(x) to realize the broadcast authentication of the group members, and each member can confirm the source and integrity of the broadcast information from BS or cluster heads.
- Session key. Session key information is encrypted by the former authentication key to ensure the security of intermediate interactive information. Using the initial private function g(x), a multiple asymmetric quadratic polynomial, to establish a session key management scheme, which can guarantee the independence of session key and network connectivity.
- Group key. In order to realize the secure broadcast of the sharing information among the group members in a cluster, the group key should be established at the basis of the former session key, in which cluster is the most natural communication group. Since the generation of group keys needs the joint participation of all group nodes or the associated nodes, there is a single point failure problem. According to the former two kinds of key, a group key scheme based on Lagrange interpolation polynomial is established, in which the nodes of the cluster are not directly involved.
- Network key. Network key is the communication key shared by BS and other network nodes and the generation and management scheme of network key is similar with the group key, in which the establishment idea of network key is to regard the BS and all cluster heads as a group, so network keys based on Lagrange interpolation polynomial can also be established.
- Personal key. The key of personal key establishment is to keep the privacy and independence of the key. The generation and management scheme of personal keys is also similar to the situation of group keys, the difference being that personal keys can be obtained by cluster nodes through getting the Lagrange interpolation polynomial coefficients based on their own random key information, in which the coefficients can only be obtained by corresponding nodes. The independent coefficient is defined as the personal key which only can be known by BS and the corresponding node.
- Reverse authentication. Based on the personal key to achieve one-to-one private communication, BS can verify the identity of each node, which is called the reverse authentication.

### 1.9. Organization

The paper is organized as follows: In Section 2, we analyze the characteristics of the Fourier series, quadratic polynomial, and Lagrange interpolation polynomial. In Section 3, we discuss the specific building process of five keys in LCKMS-QPLIP. In Section 4, the discuss the method for updating the five keys updating. In Section 5, we present a security analysis to verify the efficiency of LCKMS-QPLIP. In Section 6, conclusions are given.

## 2. Related Work

### 2.1. Characteristics of the Fourier Series

**Definition** **1.** *Assume that* f(x) *is a continuous and periodic function and the period is* T *. If* f(x) *satisfies the following condition:*

(1) $$f\left(x\right)=A_{0}+\overset{\infty }{\underset{n=1}{\sum }}A_{n}\sin\left(n\omega x+\varphi_{n}\right)=A_{0}+\overset{\infty }{\underset{n=1}{\sum }}\left(a_{n}\cos n\omega x+b_{n}\sin n\omega x\right)$$

*Equation (1) is called the Fourier series of the continuous function*f(x).

**Corollary** **1.** *Assuming that* f(x) *can be expanded into a uniformly convergent trigonometric series as follows:*

(2) $$f\left(x\right)=\frac{a_{0}}{2}+\overset{\infty }{\underset{k=1}{\sum }}\left(a_{k}\cos kx+b_{k}\sin kx\right)$$

(3) $$\{\begin{array}{l} a_{0}=\frac{1}{\pi }\int_{-\pi }^{\pi }f\left(x\right)dx\\ a_{k}=\frac{1}{\pi }\int_{-\pi }^{\pi }f\left(x\right)\cos kxdx,\left(k=0,1,2,\ldots \right)\\ b_{k}=\frac{1}{\pi }\int_{-\pi }^{\pi }f\left(x\right)\sin kxdx,\left(k=0,1,2,\ldots \right) \end{array}$$

**Proof.**  Firstly, by integrating both sides of Equation (2) in the range [−π,π]:

(4) $$\int_{-\pi }^{\pi }f\left(x\right)dx=\frac{a_{0}}{2}.2\pi =a_{0}\pi$$

(5) $$a_{0}=\frac{1}{\pi }\int_{-\pi }^{\pi }f\left(x\right)dx$$

Secondly, assuming n is a positive integer, multiplying cosnx and integrating in [−π,π] both sides of Equation (2):

(6) $$\begin{array}{ll} \int_{-\pi }^{\pi }f\left(x\right)\cos nxdx & =\frac{a_{0}}{2}\int_{-\pi }^{\pi }\cos nxdx\\ & +\overset{\infty }{\underset{k=1}{\sum }}\left(a_{k}\int_{-\pi }^{\pi }\cos kx\cos nxdx+b_{k}\int_{-\pi }^{\pi }\sin kx\cos nxdx\right)\\ & =\int_{-\pi }^{\pi }a_{n}\cos^{2}nxdx=a_{n}\pi \end{array}$$

(7) $$a_{n}=\frac{1}{\pi }\int_{-\pi }^{\pi }f\left(x\right)\cos nxdx$$

(8) $$b_{n}=\frac{1}{\pi }\int_{-\pi }^{\pi }f\left(x\right)\sin nxdx$$

Therefore, Corollary 1 is proved. □

### 2.2. Characteristic of Quadratic Polynomial

**Definition 2.**  *Assumethat*$f\left(x_{1},x_{2},\ldots ,x_{n}\right)$*is a multivariateand asymmetric quadratic polynomial of the real fields*P*as follows:*

(9) $$\begin{array}{cc} f\left(x_{1},x_{2},\ldots ,x_{n}\right) & =a_{11}x_{1}^{2}+a_{12}x_{1}x_{2}+\ldots +a_{1n}x_{1}x_{n}+a_{21}x_{2}x_{1}\\ & +a_{22}x_{2}^{2}+\ldots +a_{2n}x_{2}x_{n}\ldots \ldots +a_{n1}x_{n}x_{1}+a_{n2}x_{n}x_{2}+\ldots +a_{nn}x_{n}^{2}\\ & =\left(x_{1},x_{2},\ldots ,x_{n}\right)\left[\begin{array}{c} a_{11}\;\;a_{12}\;\;\ldots \;\;a_{1n}\\ a_{21}\;\;a_{22}\;\;\ldots \;\;a_{2n}\\ \ldots \ldots \ldots \ldots \ldots \ldots \\ a_{n1}\;\;a_{n2}\;\;\ldots \;\;a_{nn} \end{array}\right]\left[\begin{array}{c} x_{1}\\ x_{2}\\ \ldots \\ x_{n} \end{array}\right]=X^{T}AX \end{array}$$

*where A is called the quadratic matrixA of*$f\left(x_{1},x_{2},\ldots ,x_{n}\right)$*, and*$a_{ij}=a_{ji},i,j=1,\ldots ,n$*, which shows that A is a symmetric matrix or*$A=A^{T}$.

**Definition 3.**  *Assumingthat A is the quadraticmatrix of*$f\left(x_{1},x_{2},\ldots ,x_{n}\right)$*in fields*P*, if there is a non-zero real vector*ξ*coupling with a real number*λ*of fields*P*and they satisfy the function*Aξ=λξ*, in which*λ*is called the eigenvalue of matrix A and*ξ*is called the eigenvector of*λ.

It can be concluded that (λE−A)ξ=0 based on Aξ=λξ, which also indicates that ξ is a non-zero solution of Equation (10). The necessary and sufficient condition for a non-zero solution is that ξ satisfies the equation |λE−A|=0:

(10) $$\{\begin{array}{l} \left(\lambda -a_{11}\right)x_{1}-a_{12}x_{2}-\ldots -a_{1n}x_{n}=0\\ -a_{21}x_{1}+\left(\lambda -a_{22}\right)x_{2}-\ldots -a_{2n}x_{n}=0\\ \ldots \ldots \ldots \ldots \ldots \ldots \ldots \ldots \ldots \ldots \ldots \ldots \ldots \\ -a_{n1}x_{1}-a_{n2}x_{2}-\ldots +\left(\lambda -a_{nn}\right)x_{n}=0 \end{array}$$

(11) $$\left|\lambda E-A\right|=\left|\begin{array}{l} \lambda -a_{11}-a_{12}\ldots -a_{1n}\\ -a_{21}\lambda -a_{22}\ldots -a_{2n}\\ \ldots \ldots \ldots \ldots \ldots \\ -a_{n1}-a_{n2}\ldots \lambda -a_{nn} \end{array}\right|$$

where |λE−A| is called the characteristic polynomial of matrix A.

Therefore, based on Definitions 2 and 3, the method to obtain the eigenvalues and eigenvectors of matrix A can be divided into the following steps:

Step 1: In fields P, choosing a multivariate and asymmetric quadratic polynomial $f\left(x_{1},x_{2},\ldots ,x_{n}\right)$ randomly and writing out the matrix A.
Step 2: Calculating the all roots of the characteristic equation |λE−A|=0 in fields P which are also called eigenvalues.
Step 3: Taking the obtained eigenvalues into Equation (10) one by one, and then working out a group of basic solutions for each eigenvalue which are called the linearly independent eigenvectors of each eigenvalue. Therefore, based on this method, all linearly independent eigenvectors belonged to each eigenvalue can be obtained.

**Theorem 1.**  *If matrix A is a realsymmetricmatrix, for any two non-zero vectors*α,β*in fields*P*, it can be proved that*(Aα,β)=(α,Aβ).

**Proof.**  Actually, it is easy to obtain that:

(12) $$\left(A\alpha ,\beta \right)=\beta^{T}\left(A\alpha \right)=\beta^{T}A^{T}\alpha ={\left(A\beta \right)}^{T}\alpha =\alpha^{T}\left(A\beta \right)=\left(\alpha ,A\beta \right)\;$$

Therefore, (Aα,β)=(α,Aβ) and this property of the symmetric matrix *A* is also called symmetric transformation. □

**Theorem 2.**  *If matrix A is a real symmetric matrix, any two non-zero eigenvectors belonged to different eigenvalues of A in fields P must be orthogonal.*

**Proof.**  Assuming that λ,μ are the different eigenvalues of *A* and α,β are respectively belonged to λ,μ, and Aα=λα,Aβ=μβ:

Based on Theorem 1, (Aα,β)=(α,Aβ).

Therefore, (Aα,β)=(λα,β)=(α,Aβ)=(α,μβ).

And, (λα,β)=(α,μβ)⇒λ(α,β)=μ(α,β).

If λ≠μ, then (α,β)=0.

Therefore, it is shown that any two non-zero eigenvectors belonging to different eigenvalues of *A* in fields P must be orthogonal. □

**Theorem 3.**  *For any real symmetric matrix A, therewillbe a orthogonal matrix B and*$B^{T}AB=B^{-1}AB=C$*is a diagonal matrix, where*$B^{T}=B^{-1}$*and*$B^{T}B=E$.

**Proof.**  To prove the existence of matrix *B*, supposing *B* is composed of the eigenvectors $\left\{\xi_{1},\xi_{2},\ldots ,\xi_{n}\right\}$ of matrix *A* or $B=\left[\xi_{1},\xi_{2},\ldots ,\xi_{n}\right]$:

(13) $$\begin{array}{c} B^{T}AB={\left[\xi_{1},\xi_{2},\ldots ,\xi_{n}\right]}^{T}A\left[\xi_{1},\xi_{2},\ldots ,\xi_{n}\right]={\left[\xi_{1},\xi_{2},\ldots ,\xi_{n}\right]}^{T}\left[A\xi_{1},A\xi_{2},\ldots ,A\xi_{n}\right]\;={\left[\xi_{1},\xi_{2},\ldots ,\xi_{n}\right]}^{T}\left[\lambda_{1}\xi_{1},\lambda_{2}\xi_{2},\ldots ,\lambda_{n}\xi_{n}\right]\\ =\left[\begin{array}{l} \xi_{1}{}^{T}\lambda_{1}\xi_{1}\xi_{1}{}^{T}\lambda_{2}\xi_{2}\ldots \xi_{1}{}^{T}\lambda_{n}\xi_{n}\\ \xi_{2}{}^{T}\lambda_{1}\xi_{1}\xi_{2}{}^{T}\lambda_{2}\xi_{2}\ldots \xi_{2}{}^{T}\lambda_{n}\xi_{n}\\ \ldots \ldots \ldots \ldots \\ \xi_{n}{}^{T}\lambda_{1}\xi_{1}\xi_{n}{}^{T}\lambda_{2}\xi_{2}\ldots \xi_{n}{}^{T}\lambda_{n}\xi_{n} \end{array}\right]\\ =C \end{array}$$

For matrix C, if $B=\left[\xi_{1},\xi_{2},\ldots ,\xi_{n}\right]$ is a orthogonal matrix, then $\left(\xi_{i},\xi_{j}\right)=0,$ where i,j=1…n,i≠j:

(14) $$C=\left[\begin{array}{l} \xi_{1}{}^{T}\lambda_{1}\xi_{1}0\ldots 0\\ 0\xi_{2}{}^{T}\lambda_{2}\xi_{2}\ldots 0\\ \ldots \ldots \ldots \ldots \\ 00\ldots \xi_{n}{}^{T}\lambda_{n}\xi_{n} \end{array}\right]$$

Therefore, for satisfying Equation (14), $\left(\xi_{i},\xi_{j}\right)=0$ is the necessary condition, where i,j=1…n,i≠j.

According to Theorem 2, any two non-zero eigenvectors belonging to different eigenvalues of A in fields P must be orthogonal, so the current problem is to make the eigenvectors belonging to the same eigenvalue of matrix A orthogonal.

In order to achieve orthogonalization, the Gram-Schmidt orthogonalization method is applied. The process of Gram Schmidt orthogonalization is as follows: Assume that the initial vector group is $\left\{\alpha_{1},\alpha_{2},\ldots ,\alpha_{n}\right\}$, and assume:

(15) $$\begin{array}{l} \beta_{1}=\alpha_{1},\eta_{1}=\frac{\beta_{1}}{\beta_{1}},\\ \beta_{2}=\alpha_{2}-\left(\alpha_{2},\eta_{1}\right)\eta_{1}\eta_{2}=\frac{\beta_{2}}{\beta_{2}},\\ \beta_{3}=\alpha_{3}-\left(\alpha_{3},\eta_{1}\right)\eta_{1}-\left(\alpha_{3},\eta_{2}\right)\eta_{2}\eta_{2}=\frac{\beta_{2}}{\beta_{2}},\\ \ldots \ldots \ldots \ldots \\ \beta_{n}=\alpha_{n}-\overset{n-1}{\underset{i=1}{\sum }}\left(\alpha_{n},\eta_{i}\right)\eta_{i}\eta_{n}=\frac{\beta_{n}}{\beta_{n}}. \end{array}$$

where $\beta_{i},i=1,\ldots ,n$ represents the mod of the orthogonal vector $\beta_{i}$ and $\left(\alpha_{n},\eta_{i}\right)$ represents the inner product of these two vectors.

In this way, the orthogonal vector group $\left\{\beta_{1},\beta_{2},\ldots ,\beta_{n}\right\}$ of $\left\{\alpha_{1},\alpha_{2},\ldots ,\alpha_{n}\right\}$ is obtained and $\left\{\eta_{1},\eta_{2},\ldots ,\eta_{n}\right\}$ is the unit standard orthogonal vector group.

So, based on the method of Gram-Schmidt orthogonalization, these different eigenvectors belonging to the same eigenvalues of matrix $B=\left[\xi_{1},\xi_{2},\ldots ,\xi_{n}\right]$ are converted into the unit standard orthogonal vectors which compose the unit orthogonal matrix $B^{\prime }=\left[\xi_{1}{}^{\prime },\xi_{2}{}^{\prime },\ldots ,\xi_{n}{}^{\prime }\right]$, where $\left(\xi_{i}{}^{\prime },\xi_{i}{}^{\prime }\right)=1,\left(\xi_{i}{}^{\prime },\xi_{j}{}^{\prime }\right)=0,$ i≠j, i,j=1,…,n. If assume $B=B^{\prime }=\left[\xi_{1}{}^{\prime },\xi_{2}{}^{\prime },\ldots ,\xi_{n}{}^{\prime }\right]$, then:

(16) $$C=\left[\begin{array}{l} \lambda_{1}0\ldots 0\\ 0\lambda_{2}\ldots 0\\ \ldots \ldots \ldots \ldots \ldots \\ 00\ldots \lambda_{n} \end{array}\right]$$

Therefore, based on the above proof, for any real symmetric matrix A, there will actually be a unit orthogonal matrix $B=\left[\xi_{1}{}^{\prime },\xi_{2}{}^{\prime },\ldots ,\xi_{n}{}^{\prime }\right]$ and $B^{T}AB=B^{-1}AB=C$ is a diagonal matrix, where the diagonal values are the eigenvalues of the matrix A. □

**Corollary 1.**  *Any quadratic polynomial* $f\left(x_{1},x_{2},\ldots ,x_{n}\right)$ *in real field can be transformed into the sum of squares by orthogonal linear substitution, where the sum can be written as* $\lambda_{1}y_{1}^{2}+\lambda_{2}y_{2}^{2}+\ldots +\lambda_{n}y_{n}^{2}$ *and* $\lambda_{1},\lambda_{2},\ldots ,\lambda_{n}$ *are the eigenvalues of the matrix A.*

To sum up, the method of realizing orthogonal diagonalization of matrix A can be divided into the following steps:

Step 1: In fields P, selecting a quadratic polynomial $f\left(x_{1},x_{2},\ldots ,x_{n}\right)$ randomly and building matrix A, calculating all eigenvalues $\lambda_{1},\lambda_{2},\ldots ,\lambda_{n}$ and eigenvectors $\left\{\xi_{1},\xi_{2},\ldots ,\xi_{n}\right\}$ of the characteristic equation |λE−A|=0 in fields P.
Step 2: Using the method of Gram-Schmidt Orthogonalization to orthogonalize the eigenvectors $\left\{\xi_{1},\xi_{2},\ldots ,\xi_{n}\right\}$ and get the unit orthogonal matrix $B=\left[\xi_{1}{}^{\prime },\xi_{2}{}^{\prime },\ldots ,\xi_{n}{}^{\prime }\right]$, where $B^{T}=B^{-1}$ and $B^{T}B=E$.
Step 3: Based on orthogonal linear substitution $B^{T}AB=C$, matrix A can be converted into the diagonal matrix C, where the diagonal values of C are the eigenvalues of the matrix A. At the same time, realizing the linear standardization $f\left(y_{1},y_{2},\ldots ,y_{n}\right)=\lambda_{1}y_{1}^{2}+\lambda_{2}y_{2}^{2}+\ldots +\lambda_{n}y_{n}^{2}$ from $f\left(x_{1},x_{2},\ldots ,x_{n}\right)$.

### 2.3. Lagrange Interpolation Polynomial

**Definition 4.**  *Based on the uniqueness of the n-th interpolation polynomial, defining the corresponding n-th interpolation basis function*$l_{i}\left(x\right)$*for each interpolation point*$x_{i}$*, where there are*n+1*different interpolation points*$x_{i},i=0,1,2,\ldots ,n$.

Set that $x_{0},x_{1},\ldots ,x_{i-1},x_{i+1},\ldots ,x_{n}$ are the zero points of function $l_{i}\left(x\right)$ and assuming that:

(17) $$l_{i}\left(x\right)=a_{i}\left(x-x_{0}\right)\left(x-x_{1}\right)\ldots \left(x-x_{i-1}\right)\left(x-x_{i+1}\right)\ldots \left(x-x_{n}\right)$$

If setting $l_{i}\left(x\right)=1$ and $x=x_{i}$, then:

(18) $$l_{i}\left(x_{i}\right)=a_{i}\left(x_{i}-x_{0}\right)\left(x_{i}-x_{1}\right)\ldots \left(x_{i}-x_{i-1}\right)\left(x_{i}-x_{i+1}\right)\ldots \left(x_{i}-x_{n}\right)=1$$

(19) $$a_{i}=\frac{1}{\left(x_{i}-x_{0}\right)\left(x_{i}-x_{1}\right)\ldots \left(x_{i}-x_{i-1}\right)\left(x_{i}-x_{i+1}\right)\ldots \left(x_{i}-x_{n}\right)}$$

therefore:

(20) $$l_{i}\left(x\right)=\frac{\left(x-x_{0}\right)\left(x-x_{1}\right)\ldots \left(x-x_{i-1}\right)\left(x-x_{i+1}\right)\ldots \left(x-x_{n}\right)}{\left(x_{i}-x_{0}\right)\left(x_{i}-x_{1}\right)\ldots \left(x_{i}-x_{i-1}\right)\left(x_{i}-x_{i+1}\right)\ldots \left(x_{i}-x_{n}\right)}$$

and set:

(21) $$L_{n}\left(x\right)=\overset{n}{\underset{i=0}{\sum }}l_{i}\left(x\right)f\left(x_{i}\right)$$

It is shown in Equation (21) that the degree of $L_{n}\left(x\right)$ is less than n, and $L_{n}\left(x_{i}\right)=f\left(x_{i}\right),i=0,1,2,\ldots ,n$. Therefore, $L_{n}\left(x\right)$ is the interpolation polynomial for $x_{0},x_{1},\ldots ,x_{n}$ which is known as the Lagrange interpolation polynomial.

**Corollary 2.**  *Lagrange interpolation polynomial is a special form of the Chinese Remainder Theorem.*

**Proof.**  Based on the definition of Chinese Remainder Theorem [40], assuming that $m_{1}\left(x\right),m_{2}\left(x\right),\ldots ,m_{n}\left(x\right)$ are pair-wise coprime polynomials, where $a_{1}\left(x\right),a_{2}\left(x\right),\ldots ,a_{n}\left(x\right)$ are all polynomials of x, and there will be a polynomial f(x):

(22) $$\{\begin{array}{l} f\left(x\right)\equiv a_{1}\left(x\right)\left(\mathrm{mod}m_{1}\left(x\right)\right)\\ f\left(x\right)\equiv a_{2}\left(x\right)\left(\mathrm{mod}m_{2}\left(x\right)\right)\\ \ldots \ldots \\ f\left(x\right)\equiv a_{n}\left(x\right)\left(\mathrm{mod}m_{n}\left(x\right)\right) \end{array}$$

The form of f(x) is unique when the degree of f(x) is less than M(x), where $M\left(x\right)=m_{1}\left(x\right)m_{2}\left(x\right)\ldots m_{r}\left(x\right)$.

Specially, when $m_{i}\left(x\right)=x-b_{i}\in Q\left[x\right]$ (or R[x]), i=1,2,…,n, $b_{i}\left(i=1,2,\ldots ,n\right)$ are constant and not equal each other, and $m_{i}\left(x\right)\left(i=1,2,\ldots ,n\right)$ are also pair-wise coprime polynomials, so based on the Remainder Theorem, $m_{i}\left(x\right)\equiv m_{i}\left(b_{i}\right)\left(\mathrm{mod}\left(x-b_{i}\right)\right)$.

Corollary 2 can be expressed by stating that there will be a polynomial f(x):

(23) $$\{\begin{array}{l} f\left(x\right)\equiv a_{1}\left(x\right)\left(\mathrm{mod}\left(x-b_{1}\right)\right)\\ f\left(x\right)\equiv a_{2}\left(x\right)\left(\mathrm{mod}\left(x-b_{2}\right)\right)\\ \ldots \ldots \\ f\left(x\right)\equiv a_{n}\left(x\right)\left(\mathrm{mod}\left(x-b_{n}\right)\right) \end{array}\;$$

The form of f(x) is unique when the degree of f(x) is less than n, where $a_{i}\left(x\right)$ (i=1,2,…,n) are random constant.

Because $f\left(x\right)\equiv a_{i}\left(\mathrm{mod}\left(x-b_{i}\right)\right)$ is equivalent to $f\left(b_{i}\right)\equiv a_{i}$ (i=1,2,…,n), for any different $b_{i}$ (i=1,2,…,n), there will be a unique f(x) which degree is less than n. It is the reason of the existence and uniqueness of interpolation polynomial.

According to the proof of Corollary 2, there is a polynomial $M_{i}\left(x\right)$ (i=1,2,…,n), and:

(24) $$\{\begin{array}{l} M_{i}\left(x\right)\equiv 1\left(\mathrm{mod}\left(x-b_{i}\right)\right)\\ M_{j}\left(x\right)\equiv 0\left(\mathrm{mod}\left(x-b_{j}\right)\right) \end{array},i\neq j$$

Since $M_{i}\left(x\right)=\frac{\left(x-b_{1}\right)\cdots \left(x-b_{i-1}\right)\left(x-b_{i+1}\right)\ldots \left(x-b_{n}\right)}{\left(b_{i}-b_{1}\right)\cdots \left(b_{i}-b_{i-1}\right)\left(b_{i}-b_{i+1}\right)\ldots \left(b_{i}-b_{n}\right)}$ can satisfy Equation (24), interpolation polynomial f(x) can be like as:

(25) $$f\left(x\right)=a_{1}M_{1}\left(x\right)+a_{2}M_{2}\left(x\right)+\cdots +a_{n}M_{n}\left(x\right)=\overset{n}{\underset{j=1}{\sum }}a_{j}\overset{n}{\underset{i=1}{\prod }}\frac{\left(x-b_{i}\right)}{\left(b_{j}-b_{i}\right)}\left(i\neq j\right)$$

It is clear from Equation (25) that f(x) is the famous Lagrange interpolation polynomial which also is a special form of the Chinese Remainder Theorem. □

## 3. LCKMS-QPLIP

### 3.1. Network Model

**Figure 1:**
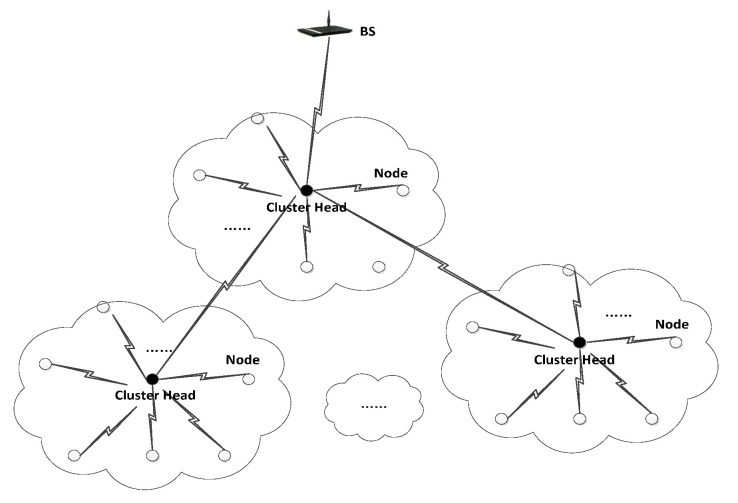
WSN layer-cluster network structure.

To facilitate the discussion, the network model of LCKMS-QPLIP is assumed as follows:

(1) It is assumed that the network is homogeneous and static, and each group member is identical in the configuration of hardware and software, where the network size is N and there are three types of nodes: base station, cluster head and common sensor node. The layer-cluster network structure of WSN shown in the Figure 1.
(2) It is assumed that BS is equipped with sufficient hardware and software resources and has stored the basic information of all nodes in the network. In addition, BS can detect the broken or captured nodes.
(3) The cluster head is responsible for collecting the data from its members and sending it to BS layer by layer. The clustering protocol LEACH [41] in WSN is chosen to initialize the network topology and select the cluster heads in this paper.
(4) The common sensor nodes are responsible for collecting the surrounding environment data and sending the data to their neighbor nodes or cluster head. Common sensor nodes have not enough storage space and energy to process data. Since the communication radius of common sensor nodes is limited, the communication between nodes that are not within the communication radius needs to rely on the transit of their common neighbor nodes.

The explanation of main symbols is shown in Table 1:

### 3.2. Building Layer-Cluster Key

Based on the idea of LEAP protocol which relies on the master key to build the main four different keys (including individual key, session key, group key and cluster key), this paper will study and design a new wireless sensor network layer-cluster key management scheme according to the requirement of the WSN security communication process.

Unlike LEAP which depends on a master key and suffers from the single-point failure problem, the new key management scheme named LCKMS-QPLIP is based on the mathematical characteristics of the quadratic polynomial and Lagrange interpolation polynomial, in which it includes five different keys (including broadcast authentication key, session key, group key, network key and personal key).

The most obvious features of this scheme compared with LEAP are the identity authentication and the independence of each key. The following will be described in sequence according to the keys’ building order in LCKMS-QPLIP.

#### 3.2.1. Forward Broadcast Authentication Key Management

The establishment of broadcast authentication key is the most obvious difference between LCKMS-QPLIP and LEAP, which is the first step of key management and the first barrier of WSN security.

Broadcasting is the most important way of data transmission in wireless networks, including command transmission from BS, information exchange between neighbor nodes, network updating, and so on. Broadcast messages without security mechanisms are vulnerable to be eavesdropped, tampered, and forged, which threatens WSN heavily, so broadcast authentication is one of the most basic security services in wireless sensor networks.

The security guarantee provided by broadcast authentication for broadcast message is consistent with the process of general message authentication, including two aspects: one is to ensure the legitimacy of the message source, and the other is to ensure the integrity of the message. Based on the broadcast authentication protocol, the receiving nodes can filter out the tampered and forged broadcast messages and ensure that the data received by the user is true and valid.

To sum up, broadcast authentication is a process of key management. While, for realizing the secure broadcasting communication management of WSN, the first thing to do is to realize the authentication between nodes.

The scheme flow of generation and management of forward broadcast authentication key is as follows:

(1) The generation of inner-cluster broadcast authentication key based on a Fourier series

The purpose of an authentication key is to realize the authentication of the source and the integrity of the broadcast message. It is assumed that the authentication key is $\;K_{i}\;$ and f(x) is a continuous and integrable function in the real field [−π,π] which also satisfies the conditions of a Fourier series. In addition, assuming that each WSN node is preset with two functions at the network initialization including a sharing function f(x) and a private function g(x). It should be noted that the private function g(x) of each node is different and each cluster shares a different sharing function f(x).

Based on [42] proposed by the first author, it is assumed that BS divides the network time into equal time slice D and allocates an independent key separately for each time slice, where the authentication key assigned to the *i*-th time slice is:

(26) $$K_{i}=\frac{a_{0}}{2}+\overset{i}{\underset{k=1}{\sum }}\left(a_{k}\cos kx+b_{k}\sin kx\right)$$

(27) $$K_{i+1}=\frac{a_{0}}{2}+\overset{i+1}{\underset{k=1}{\sum }}\left(a_{k}\cos kx+b_{k}\sin kx\right)=K_{i}+\left(a_{i+1}\cos(i+1)x+b_{i+1}\sin(i+1)x\right)$$

Obviously, according to Equation (27), the key of each time slice is different and the common node only needs to calculate the coefficients $a_{i+1}$ and $b_{i+1}$ combined with the former authentication $K_{i}$ to work out the authentication key $K_{i+1}$ of the (i+1)−th time slice.

Then, BS generates the broadcast authentication information L(i) and broadcasts it:

(28) $$\;L\left(i\right)=\{P_{i\left(t\right)}\left|\left|h\left(a_{i}\right)\right|\right|h\left(b_{i}\right)\left|\left|MAC=h\left(K_{i},P_{i\left(t\right)},i\left(t\right)\right)\right|\right|i\left(t\right)$$

where $a_{i}=\frac{1}{\pi }\int_{-\pi }^{\pi }f\left(x\right)\cos ixdx$, $b_{i}=\frac{1}{\pi }\int_{-\pi }^{\pi }f\left(x\right)\sin ixdx$, and $a_{i}$, $b_{i}$ are two Fourier series coefficients belonged to the time slice *i*, $P_{i\left(t\right)}$ is the plaintext message at time  i(t), $MAC=h\left(K_{i},P_{i\left(t\right)},i\left(t\right)\right)$ guarantees the privacy of $K_{i}$, i(t) is *t* time of the *i*-th time slice.

(2) Judging the timeliness of a package

Based on the broadcast authentication information L(i), if the last message time is i(t+1) and the current message time is i(t), it can be judged that the current authentication message L(i) is outdated and it is necessary to detect the local time of the node if the outdated packets appear in succession. For this problem, the receiving node will also make misjudgment and discard the all later authentication messages if the local time of the node is not adjusted in time.

Therefore, for this case, it is necessary to make periodic time synchronization and early warning judgment. In order to guarantee the key management process, this paper will use the time synchronization method proposed by the first author [43].

(3) Key authentication

After finishing the time synchronization operations, the local nodes need to make entity authentication and message source authentication according to *L*(*i*).

For entity authentication, since each node has been preset a function *f*(*x*), each local node can calculate the coefficients $a_{i}{}^{\prime }$, $b_{i}{}^{\prime }$ belonged to the current time slice i according to the Fourier series coefficient characteristics:

(29) $$\begin{array}{c} a_{i}{}^{\prime }=\frac{1}{\pi }\int_{-\pi }^{\pi }f\left(x\right)\cos ixdx\\ b_{i}{}^{\prime }=\frac{1}{\pi }\int_{-\pi }^{\pi }f\left(x\right)\sin ixdx \end{array}$$

Through *L*(*i*), the local node have obtained the hash function $h\left(a_{i}\right)$, $h\left(b_{i}\right)$ of the Fourier series coefficients $a_{i}{}^{\prime }$, $b_{i}{}^{\prime }$ belonged to the broadcast source or BS time slice i. If $h\left(a_{i}{}^{\prime }\right)=h\left(a_{i}\right)andh\left(b_{i}{}^{\prime }\right)=h\left(b_{i}\right)$, which indicates that the message is sent by the BS at the *i*-th time slice, and the entity identity authentication work is finished; otherwise, applying for the BS verification.

For message source authentication, the authentication key is used to determine whether the plaintext message $P_{i\left(t\right)}$ has been tampered, so the local node need to calculate the authentication key $K_{i}^{\prime }$ belonged to the current time slice *i*:

(30) $$K_{i}^{\prime }=\frac{a_{0}{}^{\prime }}{2}+\overset{i}{\underset{k=1}{\sum }}\left(a_{k}{}^{\prime }\cos kx+b_{k}{}^{\prime }\sin kx\right)$$

(31) $${K^{\prime }}_{i}=K_{i-1}+\left(a_{i}{}^{\prime }\cos ix+b_{i}{}^{\prime }\sin ix\right)$$

where $K_{i-1}$ is the authenticated key of the (i+1)−th time slice, and $a_{i}{}^{\prime }$, $b_{i}{}^{\prime }$ have been authenticated at Equation (29). In this way, only the current coefficients of the Fourier series are needed to be calculated and the calculation cost is much low.

Lastly, if $h\left(K_{i}{}^{\prime },P_{i\left(t\right)},i\left(t\right)\right)=h\left(K_{i},P_{i\left(t\right)},i\left(t\right)\right)=MAC$, it is indicated that *K_i_* is authenticated and the message source is also authenticated.

For layer-cluster network, if assuming that each cluster head and its group nodes of the cluster form a broadcast area, and different clusters are preset different f(x), the forward authentication of each cluster can be realized according to the above key authentication process. Meanwhile, the identity authentication between cluster head and base station can be realized by the same authentication method.

After completing all the authentication work and making sure that the network nodes are all belonged to their own network, the next work is to realize the session security between the two neighbor nodes called session key management.

#### 3.2.2. Session Key Management Scheme Based on a Quadratic Polynomial

Session keys are keys shared between neighbor nodes, which are used for the secure exchange of information between nodes. At present, E-G, q-composite and other popular WSN session key management schemes are flexible and simple, but the problems of these schemes are that the shared keys between the neighbor nodes is not unique and the network connectivity is low, so that the attackers can easily obtain key information to make various malicious attacks.

Therefore, based on the advantages of the existing symmetric polynomial key pre-distribution schemes in anti-capture and connectivity, this paper proposes a WSN session key management scheme based on multiple asymmetric quadratic polynomials, which is built to solve the problems of session key independence and network connectivity.

The generation and management processes of the session key based on Quadratic Polynomials are as follows:

(1) Initialization

Assume that BS generates a quadratic polynomial keys pool (i.e., private function pool about g(x)) during network initialization and records the identifier $ID_{i}$ of each common node of the network and the identifier $\left(ID_{i}||\omega_{i}\right)$ of the quadratic polynomial assigned to the common node each time. Each common node stores an independent quadratic polynomial $g_{\omega_{i}}\left(x_{1},x_{2},\ldots ,x_{n}\right)=X^{T}AX$.

(2) Building session key

Since the deployment area of the network is not secure, a secure link must be established between neighbor nodes to protect the possible communication. The establishment process of secure link is as follows:

- Getting neighbor list

Firstly, after the initialization and authentication of the layer-cluster network, the common nodes in each cluster begin to broadcast their own ID and receive the ID information of each neighbor node at the same time, and then establish their own neighbor list $\left(ID_{j}||ID_{k}\left|\left|\ldots \right|\right|ID_{m}\right)$.

Secondly, according to the previous authentication work, if $K_{i}=K_{i}{}^{\prime }$ in time slice i, using the authentication key $K_{i}$ of time slice *i* to encrypt the neighbor list information $E_{K_{i}}\left(ID_{i}\left|\left|ID_{j}\right|\right|ID_{k}\left|\left|\ldots \right|\right|ID_{m}\right)$, where $ID_{i}$ is the identifier of sending node *i*, $ID_{j}$ is the identifier of current cluster head node j.

Each cluster head will receive the encryption list information $E_{K_{i}}\left(ID_{i}\left|\left|ID_{j}\right|\right|ID_{k}\left|\left|\ldots \right|\right|ID_{m}\right)$. If the current time is still within the time slice i, the cluster head $CH_{j}$ will directly send $E_{K_{i}}\left(ID_{i}\left|\left|ID_{j}\right|\right|ID_{k}\left|\left|\ldots \right|\right|ID_{m}\right)$ to the upper layer. If the time has jumped to the next time slice i+1, using $K_{i}$ to decrypt the list firstly, and then using the authentication key $K_{i+1}$ of time slice i+1 to re-encrypt the neighbor list information $E_{K_{i+1}}\left(ID_{i}\left|\left|ID_{j}\right|\right|ID_{k}\left|\left|\ldots \right|\right|ID_{m}\right)$.

Last, BS can receive the neighbor list after several same steps and decrypt the list by authentication key $K_{i+k}$ of time slice i+k. If it fails to decrypt, BS will judge the situations whether time out of step or malicious intrusion.

- Building broadcast key information

Assuming that a is a common sensor node of cluster j, and calculating the matrix A of the private quadratic function $g_{w_{a}}\left(x_{1},x_{2},\ldots ,x_{n}\right)$ belonged to a according to Definition 2. Based on Definition 3, solving the eigenvalues $\lambda_{1},\lambda_{2},\ldots ,\lambda_{n}$ arranged in the order of small to large and eigenvectors $\left\{\xi_{1},\xi_{2},\ldots ,\xi_{n}\right\}$ of matrix A, and assuming matrix $D=\left[\xi_{1},\xi_{2},\ldots ,\xi_{n}\right]$. Then, according to Theorem 3, solving the unit orthogonal matrix B and diagonal matrix C, where the diagonal values are arranged in the order of eigenvalues from small to large. Last, broadcasting key information $E_{K_{i+l}}\left(f_{w_{a}}\left(x_{1},x_{2},\ldots ,x_{n}\right)\left|\left|h\left(B\right)\right|\right|h\left(C\right)||ID_{a}\right)$ to all neighbor nodes, where $K_{i+l}$ is the authentication key of time slice i+l.

- Information judgement

If the neighbor common node m has received the key information $E_{K_{i+l}}\left(f_{w_{a}}\left(x_{1},x_{2},\ldots ,x_{n}\right)\left|\left|h\left(B\right)\right|\right|h\left(C\right)||ID_{a}\right)$ broadcasted by node a, using the authentication key $K_{i+l}$ to decrypt the message and calculating the matrix *A* according to $f_{w_{a}}\left(x_{1},x_{2},\ldots ,x_{n}\right)$, and then solving the new eigenvalues $\lambda_{1}{}^{\prime },\lambda_{2}{}^{\prime },\ldots ,\lambda_{n}{}^{\prime }$ and eigenvectors $\left\{\xi_{1}{}^{\prime },\xi_{2}{}^{\prime },\ldots ,\xi_{n}{}^{\prime }\right\}$ based on Definition 3.

Because the new eigenvalues’ sequence may be inconsistent with the source node a or tampered by attacker, which will affect the correctness of the new eigenvectors. Besides, the sequence of the eigenvectors belonged to the same eigenvalue will also affect the correctness of the results. Therefore, in order to judge the correctness of the received information $f_{w_{a}}\left(x_{1},x_{2},\ldots ,x_{n}\right)$, it is required that the eigenvalues $\lambda_{1}{}^{\prime },\lambda_{2}{}^{\prime },\ldots ,\lambda_{n}{}^{\prime }$ solved by the node m should also be arranged in the order of small to large to form the diagonal matrix $C^{\prime }$.

If $C^{\prime }=C$, it is showed that the consistency of eigenvalues is ensured. Besides, solving the unit orthogonal matrix $C^{\prime }$, if $B^{\prime }=B$, it is showed that the sequence of multiple eigenvalues is consistent.

With these two conditions, the consistency of information can be judged before and after. Therefore, in order to judge whether the information $f_{w_{a}}\left(x_{1},x_{2},\ldots ,x_{n}\right)$ is tampered or not, it can be judged by the following equations:

(32) $$\{\begin{array}{l} h\left(C\right)=h\left(C^{\prime }\right)\\ h\left(B\right)=h\left(B^{\prime }\right) \end{array}$$

**Figure 2:**
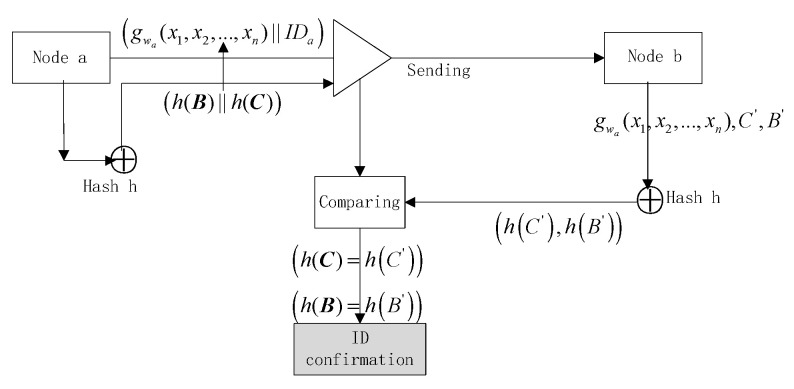
Authentication of node *a*.

The information judgment process is also equivalent to make an identity authentication of node a (as shown in Figure 2).

Based on above works, it is time to build the secure session key between node a and node m:

- Building session key

Similarly, node m broadcasts its own key information $E_{K_{i+l}}\left(f_{w_{m}}\left(x_{1},x_{2},\ldots ,x_{n}\right)\left|\left|h\left(F\right)\right|\right|h\left(G\right)||ID_{m}\right)$, and node a decrypts the key information and judges the identity of node m.

After completing the above task, the session key between two neighbor nodes can be built. In addition, the key information received by each other should be deleted to avoid information disclosure.

Assuming that the session key between node m and a is $K_{ma}=h\left(GC^{\prime }\right)$ and the session key between node a and node m is $K_{am}=h\left(CG^{\prime }\right)$. If the works of information analysis and identity judgment have been completed based on *step b* and *step c*, and then $C^{\prime }=C$, $G^{\prime }=G$, $K_{ma}=h\left(GC^{\prime }\right)=h\left(GC\right)$, $K_{am}=h\left(CG^{\prime }\right)=h\left(CG\right)$.

Because matrix C and matrix G are the standardized diagonal matrix after orthogonal, and the calculation between diagonal matrices is exchangeable, such as CG=GC. Therefore:

(33) $$K_{am}=h\left(CG\right)=h\left(GC\right)=K_{ma}$$

It is shown in Equation (33) that the only session key between node a and node m has been built, which can guarantee independence the session key for each pair neighbor nodes because of the different private quadratic polynomials belonged to the different nodes.

Considering the independence of the session key, in order to enhance the efficiency of network security management and the privacy of communication, it needs to be noted that the identity authentication key will not be used in the next steps except for keys updating.

#### 3.2.3. Group Key Management Scheme Based on Lagrange Interpolation Polynomial

Session keys can solve the problem of secure sessions between neighbor nodes, while the common communication pattern of the layer-cluster network of WSN is broadcasting in clusters, so in order to realize secure broadcasting of the shared information among the nodes in the cluster, it is necessary to set the group key based on the session key, and the cluster is the most natural communication group, so the main purpose of this part is to study and build a WSN group key management scheme based on the size of a cluster.

Group keys are the keys shared by the nodes in the same cluster, and the group keys used for encryption and decryption can only be known by the cluster members, which means that only the group members can get the encrypted message. The key point of using group keys is to solve the security problem of generation and distribution of keys.

At present, the popular group key management schemes, such as LKH and EBS, have clear structures and are easy to manage, and they support the deletion of multiple members at once. However, there are obvious problems in these schemes that the generation or acquisition of group key requires the participation of all nodes or associated nodes in the group, which is called the single point failure. In addition, that all associated nodes need to be deleted when the group key is attacked, which will influence the network structure heavily.

Therefore, the purpose of this part is to build a group key management scheme based on the above two works, identity authentication and building of session key scheme. Based on the special form Lagrange interpolation polynomial of the Chinese Remainder Theorem [40], the main idea of this scheme is that the group key can be generated without the direct participation of cluster members, which avoid the key problem of single point failure included in the above schemes [44] proposed by the first author.

The specific steps for establishing group key based on Lagrange interpolation polynomials are as follows:

Assuming that the group key of cluster j is $K_{CH_{j}}$, where the cluster head is $CH_{j}$ and the cluster size is n.

(1) Sending the key information

Firstly, each group member of cluster j generates its own key information randomly named as m(1),m(2),…,m(n), where m(i) is the key information of group member i.

Secondly, each group member encrypts its own key information by the session key generated between the group member and the cluster head independently in session key scheme. For instance, some group member i encrypts the key information m(i) by its session key $K_{i,CH_{j}}$ recorded as $E_{K_{i,CH_{j}}}\left(m\left(i\right)\right)$. After that, the group member i sends $E_{K_{i,CH_{j}}}\left(m\left(i\right)\right)$ to the cluster head $CH_{j}$.

Thirdly, the cluster head $CH_{j}$ decrypts the key information m(1),m(2),…,m(n) respectively and uses the upper layer session key ($K_{CH_{j,}CH_{k}}$ or $K_{CH_{j,}BS}$ generated between the cluster head $CH_{j}$ and the more upper layer cluster head or BS) to re-encrypt all the key information m(1),m(2),…,m(n). After that, $CH_{j}$ sends the key information $E_{K_{CH_{j},CH_{k}}}\left(m\left(1\right),m\left(2\right),\ldots ,m\left(n\right)\right)$ to BS layer by layer. In addition, every cluster head needs to delete the key information m(1),m(2),…,m(n) after the sending.

Last, BS decrypts and get the key information m(1),m(2),…,m(n).

By now, it is completed for sending the key information m(1),m(2),…,m(n) to BS.

(2) Generating Lagrange interpolation polynomial function

Firstly, BS generates a Lagrange interpolation polynomial function y(x) after getting the key information m(1),m(2),…,m(n):

(34) $$y\left(x\right)=a_{1}M_{1}\left(x\right)+a_{2}M_{2}\left(x\right)+\cdots +a_{n}M_{n}\left(x\right)=\overset{n}{\underset{j=1}{\sum }}a_{j}\overset{n}{\underset{i=1}{\prod }}\frac{\left(x-b_{i}\right)}{\left(b_{j}-b_{i}\right)}\left(i\neq j\right)$$

where $M_{i}\left(x\right)=\frac{\left(x-b_{1}\right)\cdots \left(x-b_{i-1}\right)\left(x-b_{i+1}\right)\ldots \left(x-b_{n}\right)}{\left(b_{i}-b_{1}\right)\cdots \left(b_{i}-b_{i-1}\right)\left(b_{i}-b_{i+1}\right)\ldots \left(b_{i}-b_{n}\right)}$, $m_{i}\left(x\right)=x-b_{i}\in Q\left[x\right]$ (or R[x]), i=1,2,…,n, $b_{i}\left(i=1,2,\ldots ,n\right)$ are constant and not equal each other.

Secondly, setting $b_{i}=m\left(i\right)$ and regenerating y(x) based on m(1),m(2),…,m(n), and:

(35) $$y\left(x\right)=a_{1}M_{1}^{\prime }\left(x\right)+a_{2}M_{2}^{\prime }\left(x\right)+\cdots +a_{n}M_{n}^{\prime }\left(x\right)=\overset{n}{\underset{j=1}{\sum }}a_{j}\overset{n}{\underset{i=1}{\prod }}\frac{\left(x-m\left(i\right)\right)}{\left(m\left(j\right)-m\left(i\right)\right)},\left(i\neq j\right)$$

where $M_{i}^{\prime }\left(x\right)=\frac{\left(x-m\left(1\right)\right)\cdots \left(x-m\left(i-1\right)\right)\left(x-m\left(i+1\right)\right)\ldots \left(x-m\left(n\right)\right)}{\left(m\left(i\right)-m\left(1\right)\right)\cdots \left(m\left(i\right)-m\left(i-1\right)\right)\left(m\left(i\right)-m\left(i+1\right)\right)\ldots \left(m\left(i\right)-m\left(n\right)\right)}$.

Thirdly, BS generates the group key $K_{j}$ randomly and resets a new composite function $y{\left(x\right)}^{\prime }$, and:

(36) $$y{\left(x\right)}^{\prime }=\overset{n}{\underset{j=1}{\sum }}a_{j}\overset{n}{\underset{i=1}{\prod }}\frac{\left(x-m\left(i\right)\right)}{\left(m\left(j\right)-m\left(i\right)\right)}K_{CH_{j}},\left(i\neq j\right)$$

Last, BS re-encrypts $y{\left(x\right)}^{\prime }$ by the related session key $K_{CH_{j,}BS}$ and sends it to the related cluster head $CH_{j}$.

(3) Getting the group key

Firstly, $CH_{j}$ decrypts $E_{K_{CH_{j},CH_{k}}}\left(y{\left(x\right)}^{\prime }\right)$ based on the last step.

Secondly, $CH_{j}$ sends the encrypted information $E_{K_{i,CH_{j}}}\left(y{\left(x\right)}^{\prime }\right),i=1,\ldots ,n$ to each group member.

Thirdly, node i decrypts $E_{K_{i,CH_{j}}}\left(y{\left(x\right)}^{\prime }\right)$ by $K_{i,CH_{j}}$ and gets $y{\left(x\right)}^{\prime }$.

Since:

(37) $$\{\begin{array}{l} y\left(x\right)=a_{1}M_{1}^{'}\left(x\right)+a_{2}M_{2}^{'}\left(x\right)+\cdots +a_{n}M_{n}^{'}\left(x\right)=\overset{n}{\underset{j=1}{\sum }}a_{j}\overset{n}{\underset{i=1}{\prod }}\frac{\left(x-m\left(i\right)\right)}{\left(m\left(j\right)-m\left(i\right)\right)},\left(i\neq j\right)\\ M_{i}^{'}\left(x\right)=\frac{\left(x-m\left(1\right)\right)\cdots \left(x-m\left(i-1\right)\right)\left(x-m\left(i+1\right)\right)\ldots \left(x-m\left(n\right)\right)}{\left(m\left(i\right)-m\left(1\right)\right)\cdots \left(m\left(i\right)-m\left(i-1\right)\right)\left(m\left(i\right)-m\left(i+1\right)\right)\ldots \left(m\left(i\right)-m\left(n\right)\right)} \end{array}$$

If set x=m(i), and it is concluded that:

(38) $$\{\begin{array}{l} M_{i}^{\prime }\left(m\left(i\right)\right)=1\\ M_{i}^{\prime }\left(m\left(j\right)\right)=0,i\neq j \end{array}$$

Therefore, $y\left(m\left(i\right)\right)=a_{i}$.

Similarly, $y{\left(m\left(i\right)\right)}^{\prime }=a_{i}K_{CH_{j}}$. If $a_{i}=1$, $y{\left(m\left(i\right)\right)}^{\prime }=K_{CH_{j}}$, which means that each group member can get the group key $K_{CH_{j}}$ by taking its own key information m(i) into $f{\left(x\right)}^{\prime }$ respectively.

By now, the task of getting the group key is completed. What is shown in this scheme is that the group key is generated without the direct participation of cluster members, which can solve the problem of single point failure displayed by LKH and EBS.

#### 3.2.4. Network Key Management Scheme

According to the above works, the authentication key, session key and group key have been established. Without considering the efficiency of network management, these three types of keys can basically guarantee the security of the layer-cluster network. Firstly, BS sends the information encrypted by the private session key to the neighbor cluster heads. Secondly, the first layer cluster heads re-encrypt the information and send it to the next layer cluster heads, and all the cluster heads can get the information level-by-level. Last, each cluster head uses its own group key to broadcast the information to their group members. What the problem of above scheme is that the multiple independent encryption and decryption and multi-level transmission are needed, which will cause too much computing and time cost.

According to the work of group key, if BS and all cluster heads are regarded members of a group, the base station can broadcast messages encrypted by a group key to the near cluster heads once time. If the power of the BS is large enough, all cluster heads will receive the broadcast information, and then all cluster members can receive the information encrypted by the group key belonged to different clusters.

Since this key is responsible for the broadcast information of the whole network, it is called network key $K_{N}$.

In this paper, the network key $K_{N}$ is defined as the communication key shared by the base station and all cluster head nodes, and the generation and management of the network key is similar with the group key:

(1) Each cluster head generates its own key information randomly named as m(1),m(2),…,m(r), and these cluster heads will send the key information encrypted by session keys to BS layer by layer.
(2) BS generates a Lagrange interpolation polynomial function $y{\left(x\right)}^{\prime \prime }$ after getting the key information m(1),m(2),…,m(r):
  (39) $$y{\left(x\right)}^{\prime \prime }=\overset{r}{\underset{j=1}{\sum }}a_{j}\overset{r}{\underset{i=1}{\prod }}\frac{\left(x-m\left(i\right)\right)}{\left(m\left(j\right)-m\left(i\right)\right)}K_{N},\left(i\neq j\right)$$
(3) Conversely, BS sends $y{\left(x\right)}^{\prime \prime }$ encrypted by session key to each cluster head layer by layer, and all cluster heads can obtain the network key $K_{N}$ independently based on their own key information m(i).

By now, BS can make a secure whole network broadcasting through the cooperation of $K_{N}$ and the established group key.

#### 3.2.5. Personal Key Management Scheme

These above four types of keys not only can satisfy the privacy of the information transmission, but also ensure the efficiency of network broadcasts. It is known that all the neighbor nodes communicate directly each other (including cluster head and cluster head, cluster head and BS), and the key information is encrypted or decrypted only once time between them. While there is a special situation that the communication between BS and the cluster members should be resolved and transmitted indirectly by cluster heads. It doesn’t matter if it is a broadcast information resolved and transmitted by cluster heads. But if it is a private information known only by BS and some cluster member, there will be a secure problem because of the decryption by middle cluster heads.

The requirement for personal key is usually applicable to the network with high security level and strong privacy. Therefore, in order to make the key management scheme of layer-cluster network more comprehensive and useful, the fifth key is defined as the personal key shared by common node and BS. The generation and management of personal keys is similar to that of group keys.

Assume that $K_{S_{ij},BS}$ is the personal key of BS and one common node $S_{i}$, where $S_{i}$ is one of the members of cluster j, ${CH}_{j}$ is the cluster head. The generation process of $K_{S_{ij},BS}$ is as follows:

(1) Generating Lagrange interpolation polynomial $y{\left(x\right)}^{\prime \prime \prime }$

Firstly, same as the group key, BS obtains the key information m(1),m(2),…,m(n) generated randomly by the group members of cluster j.

Secondly, BS generates the Lagrange interpolation polynomial $y{\left(x\right)}^{\prime \prime \prime }$ according to Corollary 2:

(40) $$y{\left(x\right)}^{\prime \prime \prime }=a_{1}M_{1}{}^{\prime }\left(x\right)+a_{2}M_{2}{}^{\prime }\left(x\right)+\cdots +a_{n}M_{n}{}^{\prime }\left(x\right)=\overset{n}{\underset{j=1}{\sum }}a_{j}\overset{n}{\underset{i=1}{\prod }}\frac{\left(x-m\left(i\right)\right)}{\left(m\left(j\right)-m\left(i\right)\right)}\left(i\neq j\right)$$

where $M_{i}^{\prime }\left(x\right)=\frac{\left(x-m\left(1\right)\right)\cdots \left(x-m\left(i-1\right)\right)\left(x-m\left(i+1\right)\right)\ldots \left(x-m\left(n\right)\right)}{\left(m\left(i\right)-m\left(1\right)\right)\cdots \left(m\left(i\right)-m\left(i-1\right)\right)\left(m\left(i\right)-m\left(i+1\right)\right)\ldots \left(m\left(i\right)-m\left(n\right)\right)}$.

(2) Generating key function $y{\left(x\right)}^{⁗}$

Firstly, compared with the group key, assuming that the coefficients of $y{\left(x\right)}^{\prime \prime \prime }$ are defined as $a_{i}{=K}_{S_{ij},BS}$, i=1,2,…,n. and:

(41) $$y{\left(x\right)}^{⁗}=K_{S_{1j},BS}M_{1}{}^{\prime }\left(x\right)+K_{S_{2j},BS}M_{2}{}^{\prime }\left(x\right)+\cdots +K_{S_{nj},BS}M_{n}{}^{\prime }\left(x\right)=\overset{n}{\underset{k=1}{\sum }}K_{S_{kj},BS}\overset{n}{\underset{i=1}{\prod }}\frac{\left(x-m\left(i\right)\right)}{\left(m\left(k\right)-m\left(i\right)\right)}\left(i\neq k\right)$$

Secondly, BS sends the encrypted information $E_{K_{CH_{l},BS}}\left(y{\left(x\right)}^{⁗}\right)$ to cluster head $CH_{l}$, where $K_{CH_{l},BS}$. is the session key between $CH_{l}$ and BS. With the same method, $CH_{l}$ will send the encrypted information $y{\left(x\right)}^{⁗}$ to the destination cluster node $CH_{j}$ layer by layer and $CH_{j}$ will obtain the encrypted information $E_{K_{CH_{j},CH_{k}}}\left(y{\left(x\right)}^{⁗}\right)$ at last.

Thirdly, according to the agreement built by the group key scheme, each cluster head has deleted the random key information m(1),m(2),…,m(n) after completing upward delivery. Therefore, every cluster head cannot get any useful information from $y{\left(x\right)}^{⁗}$ by m(1),m(2),…,m(n) when downward transmission of $y{\left(x\right)}^{⁗}$.

(3) Obtaining personal key

Firstly, based on above step, $CH_{j}$ has obtained $y{\left(x\right)}^{⁗}$ and then sends $E_{K_{CH_{j}}}\left(y{\left(x\right)}^{⁗}\right)$ to its cluster members, where $K_{CH_{j}}$ is the group key of cluster j.

Secondly, each cluster member can decrypt $y{\left(x\right)}^{⁗}$ by $K_{CH_{j}}$.

If x=m(i), $M_{i}^{\prime }\left(m\left(i\right)\right)=1$ and $M_{i}^{\prime }\left(m\left(j\right)\right)=0$, i≠j, and further, $y{\left(m\left(i\right)\right)}^{⁗}=a_{i}=K_{S_{ij},BS}$.

It is shown that each cluster member node can obtain its own personal key by its own random key information m(i), which can ensure the specificity and security of the personal key.

The personal key $K_{S_{ij},BS}$ can guarantee the private communication between BS and any common cluster node $S_{ij}$.

Firstly, BS encrypts the private information with the session key $K_{CH_{l},BS}$ generated with the neighbor cluster head $CH_{l}$:

(42) $$E_{K_{CH_{l},BS}}\left(E_{S_{ij},BS}\left(P\left(x\right)\right)\left|\left|ID_{j}\right|\right|E_{K_{j}}\left(ID_{i}\right)\left|\left|hash\left(ID_{j}\right)\right|\right|hash\left(P\left(x\right)\right)\right)$$

Secondly, each cluster head of the routing link can obtain the target cluster head address $ID_{j}$ from the upper cluster head and also send the private information to the next neighbor cluster head based on the neighbor list and routing table until the target cluster head $CH_{j}$ obtains the private information and verifies its identity by $hash\left(ID_{j}\right)$.

Thirdly, $CH_{j}$ obtains the final target node address $ID_{i}$ by group key $CH_{j}$ of cluster j and verifies its identity by $hash\left(ID_{i}\right)$, and then re-send the information again encrypted by session key $\;K_{i,CH_{j}}$:

(43) $$E_{K_{i,CH_{j}}}\left(E_{S_{ij},BS}\left(P\left(x\right)\right)||hash\left(P\left(x\right)\right)\right)$$

Last, $S_{i}$ obtains the plaintext information P(x) by twice decryptions with session key $\;K_{i,CH_{j}}\;$ and personal key $K_{S_{ij},BS}$, and then verifies the correction of P(x) by hash(P(x)).

Therefore, it is indicated that only BS and $S_{ij}$ can get the plaintext information P(x) in the whole private communication process.

It is known that the main function of the personal key is to guarantee the privacy of communications between each common node and BS. While, based on such one-to-one private communication, BS can verify the identity of each node which is called the reverse authentication in this paper.

Assume that the layer-cluster network needs to make a reverse authentication periodically to ensure the identity of each node, and the authentication steps are as follows:

Firstly, based on the main idea of the broadcast authentication scheme, each node uses its own private function g(x) and personal key to generate the reverse authentication information $L^{\prime }\left(i\right)$ and g(x) is a continuous and integrable function in the real field [−*π*,*π*] which also satisfy the condition of the Fourier series:

(44) $$L^{\prime }\left(i\right)=E_{K_{i,CH_{j}}}\left({\mathrm{ID}}_{BS}||E_{K_{S}{}_{ij,BS}}\left(P_{j\left(t\right)}\left|\left|h\left(a_{j}\right)\right|\right|h\left(b_{j}\right)\left|\left|h\left(E_{K_{j}}\left(P_{j\left(t\right)}\right),j\left(t\right)\right)\right|\right|j\left(t\right)\right)\right)$$

where $K_{i,CH_{j}}\;$ is the session key between $\;S_{i}\;$ and $CH_{j}$, $K_{S_{ij},BS}$ is the personal key between $\;S_{i}\;$ and BS, $K_{j}=\frac{a_{0}}{2}+\overset{j}{\underset{k=1}{\sum }}\left(a_{k}\cos kx+b_{k}\sin kx\right)$ is the authentication key allocated in the j-th time slice, $a_{j}=\frac{1}{\pi }\int_{-\pi }^{\pi }g\left(x\right)\cos ixdx$ and $b_{j}=\frac{1}{\pi }\int_{-\pi }^{\pi }g\left(x\right)\sin ixdx$ are the two Fourier coefficients of time slice j, $P_{j\left(t\right)}$ is the plaintext information of time j(t), $h\left(E_{K_{j}}\left(P_{j\left(t\right)}\right),j\left(t\right)\right)$ guarantees that $K_{j}$ is unpublished, j(t) is the time t of time slice j.

Secondly, sending $L^{\prime }\left(i\right)$, and then $CH_{j}$ decrypts $L^{\prime }\left(i\right)$ with $\;K_{i,CH_{j}}\;$ and obtains ${\mathrm{ID}}_{BS}$ which shows that $L^{\prime }\left(i\right)$ is the information for BS. After that, re-encrypting the information $L^{\prime \prime }\left(i\right)$ and sending it to the upper cluster head $CH_{l}$, where:

(45) $$L^{\prime \prime }\left(i\right)=E_{K_{CH_{j},CH_{l}}}\left({\mathrm{ID}}_{BS}||E_{K_{S}{}_{ij,BS}}\left(P_{j\left(t\right)}\left|\left|h\left(a_{j}\right)\right|\right|h\left(b_{j}\right)\left|\left|h\left(E_{K_{j}}\left(P_{j\left(t\right)}\right),j\left(t\right)\right)\right|\right|j\left(t\right)\right)\right)$$

If assuming $CH_{l}$ and BS are neighbors, and

(46) $$L^{\prime \prime \prime }\left(i\right)=E_{K_{CH_{l},BS}}\left({\mathrm{ID}}_{BS}||E_{K_{S}{}_{ij,BS}}\left(P_{j\left(t\right)}\left|\left|h\left(a_{j}\right)\right|\right|h\left(b_{j}\right)\left|\left|h\left(E_{K_{j}}\left(P_{j\left(t\right)}\right),j\left(t\right)\right)\right|\right|j\left(t\right)\right)\right)$$

Therefore, BS can decrypt $L^{\prime \prime \prime }\left(i\right)$ with $K_{CH_{l},BS}$ and learned that it is an authentication message sent by personal key.

Thirdly, for reverse authentication, entity authentication is performed first. Unlike forward authentication scheme, BS knows the private function g(x) of each node and calculates the Fourier coefficients $a_{j}{}^{\prime }$ and $b_{j}{}^{\prime }$ of current time slice j of $S_{i}$ according to the characteristics of Fourier coefficients.

(47) $$a_{j}{}^{\prime }=\frac{1}{\pi }\int_{-\pi }^{\pi }g\left(x\right)\cos ixdx,b_{j}{}^{\prime }=\frac{1}{\pi }\int_{-\pi }^{\pi }g\left(x\right)\sin ixdx$$

If $h\left(a_{j}\right)=h\left(a_{j}{}^{\prime }\right)\;\mathrm{and}\;h\left(b_{j}\right)=h\left(b_{j}{}^{\prime }\right)$, it is indicated that the message is sent by node $\;S_{i}$ at time slice j and the entity identity authentication work is completed. Otherwise, the sending node’s identity has a problem.

Last, for source authentication, it is needed to judge whether the plaintext message $P_{j\left(t\right)}$ has been tampered through the authentication key. Then, BS calculates the authentication key $K_{j}^{\prime }$ of time slice j:

(48) $$K_{j}^{\prime }=\frac{a_{0}{}^{\prime }}{2}+\overset{j}{\underset{k=1}{\sum }}\left(a_{k}{}^{\prime }\cos kx+b_{k}{}^{\prime }\sin kx\right)$$

and if $h\left(E_{K_{j}}\left(P_{j\left(t\right)}\right),j\left(t\right)\right)=h\left(E_{K_{j}{}^{\prime }}\left(P_{j\left(t\right)}\right),j\left(t\right)\right)$, it is indicated that the message sent by the $\;S_{i}\;$ is not tampered and the reverse authentication key $K_{j}$ generated by the node $\;S_{i}\;$ is correct.

By now, the identity authentication work is finished including forward authentication and reverse authentication.

To sum up, this proposed layer-cluster key management scheme of this paper guarantees the identity of network nodes through forward authentication and reverse authentication, and session key, group key and network key guarantee the security and efficiency of network, and personal key guarantees the privacy of network. These five keys complement each other, which not only ensures the independence of the keys’ management and avoids the problem of single point failure, but also enables WSN to make perform efficient key management in a reasonable network structure.

**Figure 3:**
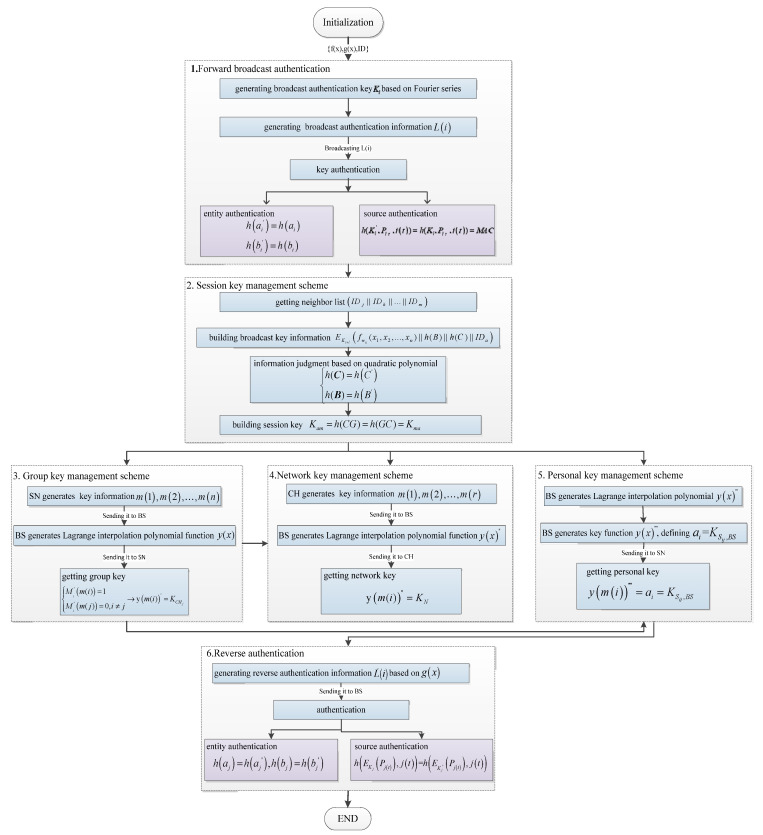
Keys association graph of layer-cluster network.

The generation principles and association of these five keys are shown in Figure 3. 

## 4. Key Updating

### 4.1. Updating f(x)

f(x) is the sharing function preset for each node during network initialization. For considering the security, f(x) needs to be updated periodically.

(1) BS generates the updating information $R_{f}\left(m\right)$.
  (49) $$R_{f}\left(m\right)=E_{K_{BS,CH_{j}}}\left(f{\left(x\right)}_{new}\left|\left|m\left(t\right)\right|\right|h\left(f{\left(x\right)}_{new}\right)||h\left(m\left(t\right)\right)\right)$$

To facilitate the discussion, assuming that BS and cluster head $CH_{j}$ are neighbors and $R_{f}\left(m\right)$ is encrypted by their session key $K_{BS,CH_{j}}$. After that, $CH_{j}$ decrypts $R_{f}\left(m\right)$ and obtains $f{\left(x\right)}_{new}$ and time slice m(t). In addition, verifying the integrity of $f{\left(x\right)}_{new}$ and the timeliness of m(t) by hash function.

(2) After verifying, $CH_{j}$ re-encrypts the updating information named $R_{f}{\left(m\right)}^{\prime }$ by group key $K_{CH_{j}}$.
  (50) $$R_{f}{\left(m\right)}^{\prime }=E_{K_{CH_{j}}}\left(f{\left(x\right)}_{new}\left|\left|m\left(t\right)\right|\right|h\left(f{\left(x\right)}_{new}\right)||h\left(m\left(t\right)\right)\right)$$

Through broadcasting, every cluster member can receive $R_{f}{\left(m\right)}^{\prime }$ and obtains $f{\left(x\right)}_{new}$ and time slice m(t) by $K_{CH_{j}}$, and also can verify the integrity of $f{\left(x\right)}_{new}$ and the timeliness of m(t) by hash function.

After the verification, each cluster member stores the new sharing function $f{\left(x\right)}_{new}$ and deletes the old sharing function f(x). According to the same method, all the network nodes can complete the updating of f(x).

### 4.2. Updating g(x)

g(x) is the private quadratic polynomial function preset for each node during network initialization, and the private function belonged to each node is different. According to the above schemes, g(x) is the key factor for the session key generation and the reverse authentication. So, the measure of updating g(x) periodically is important for network secure management.

Updating g(x) can be realized by the coordination and cooperation of BS and the personal key.

(1) Assume that BS generates the updating information $R_{g}\left(n\right)$, and $g{\left(x\right)}_{new}$ is the private function for updating:
  (51) $$R_{g}\left(n\right)=E_{K_{BS,CH_{j}}}\left(ID_{S_{ij}}\left|\left|E_{K_{S_{ij},BS}}\left(g{\left(x\right)}_{new}\left|\left|h\left(g{\left(x\right)}_{new}\right)\right|\right|h\left(ID_{S_{ij}}\right)||h\left(n\left(t\right)\right)\right)\right|\right|n\left(t\right)\right)$$

For simplicity of the discussion, also assuming that BS and cluster head $CH_{j}$ are neighbors and $R_{g}\left(n\right)$ is encrypted by their session key $K_{BS,CH_{j}}$. $CH_{j}$ can decrypt $R_{g}\left(n\right)$ and judge that $R_{g}\left(n\right)$ is the private information sent by BS at time slice n(t). After that, $CH_{j}$ will re-encrypt the updating information $R_{g}{\left(n\right)}^{\prime }$ by $K_{CH_{j},S_{ij}}$:

(52) $$R_{g}{\left(n\right)}^{\prime }=E_{K_{CH_{j},S_{ij}}}\left(ID_{S_{ij}}\left|\left|E_{K_{S_{ij},BS}}\left(g{\left(x\right)}_{new}\left|\left|h\left(g{\left(x\right)}_{new}\right)\right|\right|h\left(ID_{S_{ij}}\right)||h\left(n\left(t\right)\right)\right)\right|\right|n\left(t\right)\right)$$

(2) $S_{ij}$ decrypts $R_{g}{\left(n\right)}^{\prime }$ by $K_{CH_{j},S_{ij}}$ and judges that $R_{g}\left(n\right)$ is the private information for itself by verifying $ID_{S_{ij}}$ and n(t). After that, $S_{ij}$ continues to decrypt $g{\left(x\right)}_{new}$ by the personal key $E_{K_{S_{ij},BS}}$ and verifies the integrity of $g{\left(x\right)}_{new}$ and the timeliness of n(t) by hash function.

By this way, each cluster member node can obtain its new private function $g{\left(x\right)}_{new}$ and deletes the old one g(x).

### 4.3. Session Key Updating

As mentioned above, after the updating of g(x), each node has obtained its new privacy function $g{\left(x\right)}_{new}$. According to the session key scheme, each pair of neighbor nodes can regenerate a new session key, and the difference compared with before is that the key information is encrypted by the group key.

Assuming that the neighbor nodes a and m of cluster j are building a new session key, and the steps are as follows:

(1) Node a resolves the new private quadratic function $g_{w_{a}}{\left(x_{1},x_{2},\ldots ,x_{n}\right)}_{new}$ and gets the quadratic matrix $A_{new}$. In addition, based on Theorem 3, solving the new unit orthogonal matrix $B_{new}$, diagonal matrix $C_{new}$ and eigenvector matrix $D_{new}$, where the diagonal values are arranged in the order of eigenvalues from small to large.
(2) Broadcasting key information encrypted by group key $K_{CH_{j}}$ to all neighbor nodes:
  (53) $$E_{K_{CH_{j}}}\left(f_{w_{a}}{\left(x_{1},x_{2},\ldots ,x_{n}\right)}_{new}\left|\left|h\left(B_{new}\right)\right|\right|h\left(C_{new}\right)||ID_{a}\right)$$
(3) Information judgement. node m resolves the key information by $K_{CH_{j}}$ and gets $f_{w_{a}}{\left(x_{1},x_{2},\ldots ,x_{n}\right)}_{new}$. Based on Theorem 3, solving the unit orthogonal matrix ${B^{\prime }}_{new}$ and diagonal matrix ${C^{\prime }}_{new}$. If:
  (54) $$h\left(C_{new}\right)=h\left({C^{\prime }}_{new}\right),h\left(B_{new}\right)=h\left({B^{\prime }}_{new}\right)$$

It is indicated in Equation (54) that the key information is not tampered with and the identity of node a also is authenticated.

(4) Building the new session key. Node m also broadcasts its key information encrypted by group key $K_{CH_{j}}$ to all neighbor nodes.
  (55) $$E_{K_{CH_{j}}}\left(f_{w_{m}}{\left(x_{1},x_{2},\ldots ,x_{n}\right)}_{new}\left|\left|h\left(F_{new}\right)\right|\right|h\left(G_{new}\right)||ID_{m}\right)$$

Node a resolves the key information from m by $K_{CH_{j}}$ and judges the identity.

Therefore, defining the new session key $K_{ma}{}_{new}$ between m and a.

(56) $$K_{ma}{}_{new}=h\left(G_{new}C_{new}\right)=h\left(C_{new}G_{new}\right)=K_{am}{}_{new}$$

### 4.4. Group Key Updating

Updating of the group key is still based on the idea of Lagrange interpolation polynomial. The difference of the new key generation is that the random key information  m(1),m(2),…,m(n)  are encrypted by the personal key respectively which can guarantee that the intermediate transfer nodes or cluster nodes cannot decrypt the key information and also can guarantee the security of subsequent new network group key, network key and personal key.

The main updating ideas are as follows:

(1) Assume that $m{\left(i\right)}_{new}$ is the new key information generated by node a of cluster j, and then a encrypts $m{\left(i\right)}_{new}$ with its own personal key and the session key and sends it to cluster head $CH_{j}$, and the encrypted information is written as $E_{K_{S_{ij},CH_{j}}}\left(E_{K_{S_{ij},BS}}\left(m{\left(i\right)}_{new}\right)\right)$.
(2) $CH_{j}$ decrypts $E_{K_{S_{ij},CH_{j}}}\left(E_{K_{S_{ij},BS}}\left(m{\left(i\right)}_{new}\right)\right)$ with $K_{S_{ij},CH_{j}}$ and finds that it is a private information sent to BS. For facilitating and saving computing resources, $CH_{j}$ will wait for the all key information of the cluster members and send it to BS together (supposing $CH_{j}$ is adjacent to BS here), and the encrypted information is written as: $E_{K_{BS,CH_{j}}}\left(E_{K_{S_{1j},BS}}\left(m{\left(1\right)}_{new}\right)\left|\left|\ldots \right|\right|E_{K_{S_{ij},BS}}\left(m{\left(i\right)}_{new}\right)\left|\left|\ldots \right|\right|E_{K_{S_{nj},BS}}\left(m{\left(n\right)}_{new}\right)\right)$.
(3) BS receives and decrypts the information $m{\left(1\right)}_{new},m{\left(2\right)}_{new},\ldots ,m{\left(n\right)}_{new}$ from $CH_{j}$ by the session key $K_{BS,CH_{j}}$ and the personal keys of the members of cluster j.
(4) Generating the new group key based on the group key scheme and the steps are as follows:
  Step 1: BS generates a new Lagrange interpolation polynomial $y{\left(x\right)}^{\prime }{}_{new}=\overset{n}{\underset{j=1}{\sum }}a_{j}\overset{n}{\underset{i=1}{\prod }}\frac{\left(x-m{\left(i\right)}_{new}\right)}{\left(m{\left(j\right)}_{new}-m{\left(i\right)}_{new}\right)}K_{CH_{j}}{}_{new},\left(i\neq j\right)$, where $K_{CH_{j}}{}_{new}$ is the new group key;
  Step 2: BS encrypts $y{\left(x\right)}^{\prime }{}_{new}$, it is written as $E_{K_{BS,CH_{j}}}\left(y{\left(x\right)}^{\prime }{}_{new}\right)$ and sends it to $CH_{j}$;
  Step 3: $CH_{j}$ decrypts $y{\left(x\right)}^{\prime }{}_{new}$ and re-encrypts it with old group key, it is written as $E_{K_{CH_{j}}}\left(y{\left(x\right)}^{\prime }{}_{new}\right)$;
  Step 4: every cluster member receives the broadcast information from $CH_{j}$ and gets $y{\left(x\right)}^{\prime }{}_{new}$ by $K_{CH_{j}}$;
  Step 5: every cluster member obtains the new group key $K_{CH_{j}}{}_{new}$ by putting $m{\left(i\right)}_{new}$ into $y{\left(x\right)}^{\prime }{}_{new}$;
  Step 6: all members delete the old group key $K_{CH_{j}}$ and enable the new group key $K_{CH_{j}}{}_{new}$.

There are two obvious advantages of the group key updating scheme:

(1) $m{\left(i\right)}_{new}$ is encrypted by personal key and the intermediate transfer nodes or cluster nodes cannot obtain $m{\left(i\right)}_{new}$.
(2) $y{\left(x\right)}^{\prime }{}_{new}$ is encrypted by old group key $K_{CH_{j}}$ when it is broadcasted by cluster head, where the advantage is that the cluster members can receive the broadcast information once time and save the computing resources heavily.

In addition, $m{\left(i\right)}_{new}$ can guarantee the security of subsequent new network key and personal key.

### 4.5. Network Key Updating

The updating scheme of network key is similar with the building scheme of network key, and the specific steps are as follows:

(1) Assume that the key information $m{\left(1\right)}_{new},m{\left(2\right)}_{new},\ldots ,m{\left(r\right)}_{new}$ are generated respectively by r cluster heads and the transmitted information is encrypted by session key. In addition, for easy to discuss, it is supposed that $CH_{j}$ is adjacent to BS and encrypted information is written as $E_{K_{S_{ij},CH_{j}}}\left(m{\left(j\right)}_{new}\right)$.
(2) BS receives and decrypts the information $m{\left(1\right)}_{new},m{\left(2\right)}_{new},\ldots ,m{\left(r\right)}_{new}$ from all r cluster heads and generates a new Lagrange interpolation polynomial function $y{\left(x\right)}^{\prime \prime }{}_{new}$:
  (57) $$y{\left(x\right)}^{\prime \prime }{}_{new}=\overset{r}{\underset{j=1}{\sum }}a_{j}\overset{r}{\underset{i=1}{\prod }}\frac{\left(x-m{\left(i\right)}_{new}\right)}{\left(m{\left(j\right)}_{new}-m{\left(i\right)}_{new}\right)}K_{N}{}_{new},\left(i\neq j\right)$$
  where,$\;K_{N}{}_{new}$ is the new updating network key.
(3) BS sends $y{\left(x\right)}^{\prime \prime }{}_{new}$ to each cluster heads. The difference compared with former building scheme of network key is that $y{\left(x\right)}^{\prime \prime }{}_{new}$ is not encrypted by session key and not transmitted layer by layer, it is encrypted as $E_{K_{N}}\left(y{\left(x\right)}^{\prime \prime }{}_{new}\right)$ by the old network key $K_{N}$ and only broadcasted once time.
(4) Each cluster head obtains $y{\left(x\right)}^{\prime \prime }{}_{new}$ by $K_{N}$ after receiving $E_{K_{N}}\left(y{\left(x\right)}^{\prime \prime }{}_{new}\right)$ and then obtains the new network key $K_{N}{}_{new}$ by putting $m{\left(i\right)}_{new}$ into $y{\left(x\right)}^{\prime \prime }{}_{new}$, where the old network key $K_{N}$ will be deleted when enabling $K_{N}{}_{new}$.

To sum up, $y{\left(x\right)}^{\prime \prime }{}_{new}$ is encrypted by the old network key $K_{N}$ when it is broadcasted to all cluster heads, where the advantage is that the all cluster heads can receive the broadcast information once time and save the computing resources heavily.

### 4.6. Personal Key Updating

From those above updating schemes, personal key is the key factor to guarantee the security of other keys’ updating. So, it is very important to update the personal key.

The personal key updating scheme is similar with the building scheme of personal key, and the specific steps are as follows:

(1) According to the group key updating scheme, BS has obtained the random key information $m{\left(1\right)}_{new},m{\left(2\right)}_{new},\ldots ,m{\left(n\right)}_{new}$ of cluster j and $CH_{j}$ cannot decrypt these information. So, BS generates a new Lagrange interpolation polynomial $y{\left(x\right)}^{⁗}{}_{new}$ same as the former personal scheme procedure:
  (58) $$y{\left(x\right)}^{⁗}{}_{new}=K_{S_{1j},BS}{}_{new}M_{1}{}^{\prime }\left(x\right)+K_{S_{2j},BS}{}_{new}M_{2}{}^{\prime }\left(x\right)+\cdots +K_{S_{nj},BS}{}_{new}M_{n}{}^{\prime }\left(x\right)=\sum_{k=1}^{n}K_{S_{kj},BS}{}_{new}\prod_{i=1}^{n}\frac{\left(x-m{\left(i\right)}_{new}\right)}{\left(m{\left(k\right)}_{new}-m{\left(i\right)}_{new}\right)}\left(i\neq k\right)$$
  where $M_{i}^{\prime }\left(x\right)=\frac{\left(x-m{\left(1\right)}_{new}\right)\cdots \left(x-m{\left(i-1\right)}_{new}\right)\left(x-m{\left(i+1\right)}_{new}\right)\ldots \left(x-m{\left(n\right)}_{new}\right)}{\left(m{\left(i\right)}_{new}-m{\left(1\right)}_{new}\right)\cdots \left(m{\left(i\right)}_{new}-m{\left(i-1\right)}_{new}\right)\left(m{\left(i\right)}_{new}-m{\left(i+1\right)}_{new}\right)\ldots \left(m{\left(i\right)}_{new}-m{\left(n\right)}_{new}\right)}$, $K_{S_{ij},BS}{}_{new}$ is the new updating personal key.
(2) BS sends the encrypted information $E_{K_{CH_{j},BS}}\left(y{\left(x\right)}^{⁗}{}_{new}\right)$ to $CH_{j}$ (supposing $CH_{j}$ is adjacent to BS), where $K_{CH_{j},BS}$ is the session key. And then, $CH_{j}$ decrypts and gets $y{\left(x\right)}^{⁗}{}_{new}$, where $CH_{j}$ cannot get any useful information from $y{\left(x\right)}^{⁗}{}_{new}$ because of the lack of $m{\left(1\right)}_{new},m{\left(2\right)}_{new},\ldots ,m{\left(n\right)}_{new}$.
(3) $CH_{j}$ sends the encrypted information $E_{K_{CH_{j}{}_{new}}}\left(y{\left(x\right)}^{⁗}{}_{new}\right)$ to each cluster member of cluster j, where $K_{CH_{j}{}_{new}}$ is the new updating group key.
(4) Obtaining new personal key. $S_{ij}$ receives and obtains $y{\left(x\right)}^{⁗}{}_{new}$ by $K_{CH_{j}{}_{new}}$. If assuming $x=m{\left(i\right)}_{new}$ and putting $m{\left(i\right)}_{new}$ into $y{\left(x\right)}^{⁗}{}_{new}$, then $y{\left(m\left(i\right)\right)}^{⁗}{}_{new}=a_{i}=K_{S_{ij},BS}{}_{new}$ and the old personal key $K_{S_{ij},BS}$ will be deleted when enabling $K_{S_{ij},BS}{}_{new}$.

To sum up, it is shown that these five keys all can be updated periodically. On one hand, these updating measures can keep the freshness of keys management; on the other hand, it makes the management of key information and the establishment of new key more secure.

## 5. Security Analysis

### 5.1. Network Connectivity Analysis

Connectivity is one of the important factors of reflecting the function of the key management scheme, while the main disadvantage of popular schemes such as E-G and q-composite is that they cannot guarantee the absolute existence of shared key between any two nodes. Therefore, based on the layer-cluster network structure, the LCKMS-QPLIP scheme proposed in this paper can realize 100% secure connectivity between any pair nodes of one cluster.

For discussing the connectivity within a cluster, the main task is to build a session communication key between any non-adjacent nodes. If assuming that node a and node f are not adjacent, the specific steps of building the session key of these two nodes are as follows:

(1) Address query. node a encrypts the information $K_{S_{aj},CH_{j}}\left(ID_{a}||ID_{f}\right)$ and sends it to the cluster head $CH_{j}$, where $K_{S_{aj},CH_{j}}$ is the session key between a and $CH_{j}$.
(2) $CH_{j}$ decrypts the information and get the communication request between node a and node f. If it is queried from the neighbor list by $CH_{j}$ that node m is the common neighbor node of a and f, $CH_{j}$ will send $K_{S_{aj},CH_{j}}\left(ID_{a}\left|\left|ID_{m}\right|\right|ID_{f}\right)$ and $K_{S_{mj},CH_{j}}\left(ID_{a}\left|\left|ID_{m}\right|\right|ID_{f}\right)$ to a and f respectively which means that m is their intermediate communication node. Meanwhile, sending $K_{S_{fj},CH_{j}}\left(ID_{a}\left|\left|ID_{m}\right|\right|ID_{f}\right)$ to f which means that a and f need its help to finish the non-adjacent communication. The advantage of the above two steps is that they can reduce the probability of a cluster head $CH_{j}$ acting as the intermediate node. Actually, according to the traditional scheme, if the neighbor list of a doesn’t contain f, $CH_{j}$ has to act as the intermediate node which will increase the communication cost of $CH_{j}$. It is known that the cluster size is the one hop range of the cluster head according to de definition of layer-cluster network and the communication distance of each pair nodes in the cluster usually does not exceed 2 hops. Therefore, it is better to query and select the communication route of non-adjacent nodes by cluster head.
(3) Building the non-adjacent session key $K_{af}$. Node a sends the encrypted information $E_{K_{am}}\left(l_{a}||ID_{f}\right)$ to node m, where $l_{a}=f_{w_{a}}\left(x_{1},x_{2},\ldots ,x_{n}\right)\left|\left|h\left(B\right)\right|\right|h\left(C\right)||ID_{a}$ is the key information of node a. Node m sends the encrypted information $E_{K_{mf}}\left(l_{a}||ID_{f}\right)$ to node f. Node f decrypts and obtains $l_{a}$ and also sends $E_{K_{mf}}\left(l_{f}||ID_{a}\right)$ to node m, where $l_{f}$ is the key information of node f. Node m also sends the encrypted information $E_{K_{am}}\left(l_{f}||ID_{a}\right)$ to node a. Node a decrypts and obtains $l_{f}$. After sending the key information, node a and node f can build the non-adjacent session key $K_{af}$ based on the former session key scheme, and then node m deletes $l_{f}$ and $l_{a}$.
(4) Non-adjacent communication. Based on the non-adjacent session key $K_{af}$, node a sends the encrypted information $E_{K_{am}}\left(E_{K_{af}}\left(M\right)\left|\left|ID_{a}\right|\right|ID_{f}\right)$ to node m, where M is the plaintext. Node m decrypts the information and gets that it is the information sent to f, and then m re-encrypts the information $E_{K_{mf}}\left(E_{K_{af}}\left(M\right)\left|\left|ID_{a}\right|\right|ID_{f}\right)$ and sends it to f.

After receiving the information, node f gets that it is the information from node a and decrypts it again by $K_{af}$ to get the plaintext M.

By now, the non-adjacent communication is completed.

To sum up, there are three advantages for building the non-adjacent session key:

- The cluster head query and select the communication route of non-adjacent nodes which can reduce the communication cost.
- The intermediate node m is only responsible for forwarding the encrypted information and cannot get the plaintext, which can ensure the security of the forwarding process.
- The routing cooperation by cluster head nodes can ensure the 100% connectivity between nodes of the cluster, which is the most prominent advantage and feature of the scheme.

In addition, for realizing the non-adjacent nodes communication of different clusters, BS can act as the routing coordination node referring the above scheme, which can completely realize the secure communication of the whole network. The only difference is that the intermediate nodes need at least two cluster heads, which can increase the routing cost.

### 5.2. Security Analysis of Network Topology Change

After a period of operation, the new network will inevitably encounter two situations: one is the addition of new nodes, the other is the deletion of old nodes.

#### 5.2.1. New Node Joining

Assuming that b is the new node for joining cluster j and BS has preset ID, private quadratic polynomial function $g_{\omega_{b}}\left(x_{1},x_{2},\ldots ,x_{n}\right)$, and the sharing function f(x), group key $K_{CH_{j}}$ of current time slice of cluster j for the new node b in advance.

Firstly, node b broadcasts the encrypted information $E_{K_{CH_{j}}}\left(ID_{b}\right)$ by $K_{CH_{j}}$.

Secondly, building the neighbor list. After receiving the broadcast information, all neighbor nodes of node b in cluster j decrypt it and find that it is a new ID and not in their own neighbor list, and judge that node b is the joining node and add the new ID into their neighbor list. Similarly, node b can receive the reply information from the all neighbor nodes of cluster j, such as the reply information $E_{K_{CH_{j}}}\left(ID_{k}\right)$ of node k. And then building the neighbor list $\left(ID_{j}||ID_{k}\left|\left|\ldots \right|\right|ID_{m}\right)$ of node b and sending the encrypted information $E_{K_{CH_{j}}}\left(ID_{b}\left|\left|ID_{j}\right|\right|ID_{k}\left|\left|\ldots \right|\right|ID_{m}\right)$ to $CH_{j}$.

Thirdly, BS reorganizes the neighbor lists. $CH_{j}$ sends the encrypted information $E_{K_{CH_{j},BS}}\left(ID_{b}\left|\left|ID_{j}\right|\right|ID_{k}\left|\left|\ldots \right|\right|ID_{m}\right)$ to BS (supposing $CH_{j}$ and BS are adjacent). And then BS gets that it is the neighbor list of new joining node b. In addition, BS will add $ID_{b}$ to all neighbor lists of the neighbor nodes.

Last, building the neighbor session key. Node b establishes its own broadcast key information $E_{K_{CH_{j}}}\left(f_{w_{b}}\left(x_{1},x_{2},\ldots ,x_{n}\right)\left|\left|h\left(B\right)\right|\right|h\left(C\right)||ID_{b}\right)$ according to the quadratic polynomial $g_{\omega_{b}}\left(x_{1},x_{2},\ldots ,x_{n}\right)$ and broadcasts it. All neighbor nodes also send their own key information to node b after receiving the key information and then building the session key between new neighbors based on the former session key scheme. After building the session, node b will delete the all key information of other nodes. By now, the new node joining is completed.

To sum up, the new node joining does not affect topological structure of the network which shows the strong scalability of the scheme.

#### 5.2.2. Node Quitting

There are two situations for node quitting: one is energy exhaustion, the other is to be judged as an abnormal node.

- Energy Exhaustion Quitting

In WSN, the nodes in the high event area are often very active and their energy will be exhausted rapidly because of the high-frequency communication. For this case, when the energy of the node is close to the warning value (setting the warning value is that the left energy cannot meet the communication with the farthest neighbor node), it will notify its neighbor nodes and BS in advance, and then the node will quit the network when the energy is lower than the warning value. For this kind of node, the quitting does not affect the security of network, and the quitting scheme is relatively simple. It is assumed that node a of cluster j is about to run out of energy and quit network.

Firstly, node a periodically measures its own energy. When the energy value is close to the warning value, it will send two alarm messages to the relevant nodes: one is a broadcast message $E_{K_{CH_{j}}}\left(ID_{a}\left|\left|i\left(t\right)\right|\right|0\right)$, where i(t) is the sending time of message, 0 represents the energy warning of node a; the other is a private message $E_{K_{CH_{j},S_{ij}}}\left(ID_{BS}||E_{K_{S_{ij},BS}}\left(ID_{a}\left|\left|i\left(t\right)\right|\right|0\right)\right)$.

Secondly, all neighbor nodes (including cluster head $CH_{j}$) of node a decrypt the broadcast message and learn that it is a warning message of energy sent at time i(t), and then delete $ID_{a}$ from the neighbor lists.

Thirdly, $CH_{j}$ decrypts the private message and learns that it is a private message sent to BS, and then sends the re-encrypted information $E_{K_{CH_{j},BS}}\left(ID_{CH_{j}}\left|\left|ID_{a}\right|\right|E_{K_{S_{ij},BS}}\left(ID_{a}\left|\left|i\left(t\right)\right|\right|0\right)\right)$ to BS (supposing $CH_{j}$ and BS are adjacent).

Last, BS decrypts the private message and learns that it is a private information from node a, and then further learns that it is an energy warning message of node a sent at time i(t) sends the energy alarm information at any time. After that, BS reorganizes the neighbor lists and deletes $ID_{a}$ from the all neighbor nodes’ lists of node a, and then deletes the neighbor lists of node a.

By now, node a has quitted the network, and it can be judged directly that it is an abnormal node if the network nodes still can receive some information from node a.

- Abnormal Node Quitting

If BS has detected that node c is an abnormal node of cluster j, and it needs to cut off all the associated relationship between node c and the network. According to the proposed scheme LCKMS-QPLIP, the associated information includes sharing function f(x), session key and group key. Although the anti-capture capability of the scheme can prove that the capture of a single node will not affect the security of the network, for further security, the scheme is still designed to update the associated information including f(x), g(x), session key, group key and private key.

The updating steps are as follows:

Firstly, BS judges the abnormal behavior of node c and marks c as the quitting node.

Secondly, BS broadcasts the encrypted abnormal information $E_{K_{N}}\left(ID_{c}\left|\left|ID_{CH_{j}}\right|\right|danger\right)$ to network by $K_{N}$.

Thirdly, each cluster head decrypts the broadcast information and gets that node c is an abnormal node of cluster j, and then all cluster heads broadcast the abnormal information encrypted by their group keys to their cluster members, e.g., $E_{K_{CH_{j}}}\left(ID_{c}||danger\right)$.

Fourthly, all nodes in the network knows that node c is the abnormal quitting node, and all communication with node c is stopped, where all neighbor nodes of node c delete $ID_{c}$ from their neighbor lists and BS reorganizes the all neighbor nodes’ lists of node c after deleting $ID_{c}$.

Last, after deleting the associated information of node c, cluster j needs to update the associated information again including f(x), g(x), session key, group key and private key.

After the updating, node c will not be able to participate in any communication of the network. This quitting scheme not only implements the measures to abnormal nodes, but also lows the updating cost and keeps the updating measures in a cluster.

### 5.3. Anti-Capture Analysis

#### 5.3.1. Anti-Capture Analysis of Session Key

Since the generation of these five keys are all related the quadratic polynomial, and the building of session keys is directly generated by with quadratic polynomial, this paper will make the anti-capture analysis started with the session key.

**Corollary 3.**  *Based on the main idea of E-G scheme, the keys pool is composed of the binary t-th symmetric polynomials, and the communication of the network can be broke as long as the enemy captures t nodes containing the same polynomial, which can be called that E-G scheme only can resist t-collusion attack.*

**Proof.**  *Assuming*f(x,y) is a binary *t*-th symmetric polynomial, where:

(59) $$f\left(x,y\right)=a_{1}x^{t}+a_{2}x^{t-1}y+\ldots +a_{t-1}xy^{t-1}+a_{t}y^{t}$$

(60) $$f\left(y,x\right)=a_{1}y^{t}+a_{2}y^{t-1}x+\ldots +a_{t-1}yx^{t-1}+a_{t}x^{t}$$

According to symmetry, f(x,y)=f(y,x), and:

(61) $$a_{1}\left(x^{t}-y^{t}\right)+a_{2}\left(x^{t-1}y-y^{t-1}x\right)+\ldots +a_{t-1}\left(xy^{t-1}-yx^{t-1}\right)+a_{t}\left(y^{t}-x^{t}\right)=0$$

According to the property of symmetric polynomial, each node can calculate the session key $f\left(ID,ID^{\prime }\right)$ with other nodes who include f(x,y) based on its unique ID value. Supposing the enemy has captured t nodes with the same polynomial and the ID values are $ID_{1},ID_{2},\ldots ,ID_{t}$, every two ID are put into the Equation (61), then an t(t−1) -order polynomial group can be obtained:

(62) $$\{\begin{array}{l} a_{1}\left(ID_{1}{}^{t}-ID_{2}{}^{t}\right)+a_{2}\left(ID_{1}{}^{t-1}ID_{2}-ID_{2}{}^{t-1}ID_{1}\right)+\ldots +a_{t-1}(ID_{1}ID_{2}{}^{t-1}-ID_{2}ID_{1}{}^{t-1})+a_{t}\left(ID_{2}{}^{t}-ID_{1}{}^{t}\right)=0\\ a_{1}\left(ID_{1}{}^{t}-ID_{3}{}^{t}\right)+a_{2}\left(ID_{1}{}^{t-1}ID_{3}-ID_{3}{}^{t-1}ID_{1}\right)+\ldots +a_{t-1}(ID_{1}ID_{3}{}^{t-1}-ID_{3}ID_{1}{}^{t-1})+a_{t}\left(ID_{3}{}^{t}-ID_{1}{}^{t}\right)=0\\ \ldots \ldots \\ a_{1}\left(ID_{t}{}^{t}-ID_{t-1}{}^{t}\right)+a_{2}\left(ID_{t}{}^{t-1}ID_{t-1}-ID_{t-1}{}^{t-1}ID_{t}\right)+\ldots +a_{t-1}(ID_{t}ID_{t-1}{}^{t-1}-ID_{t-1}ID_{t}{}^{t-1})+a_{t}\left(ID_{t-1}{}^{t}-ID_{t}{}^{t}\right)=0 \end{array}$$

(63) $$[\begin{array}{l} ID_{1}{}^{t}-ID_{3}{}^{t}ID_{1}{}^{t-1}ID_{2}-ID_{2}{}^{t-1}ID_{1}\ldots ID_{1}ID_{2}{}^{t-1}-ID_{2}ID_{1}{}^{t-1}\;ID_{2}{}^{t}-ID_{1}{}^{t}\\ ID_{1}{}^{t}-ID_{3}{}^{t}ID_{1}{}^{t-1}ID_{3}-ID_{3}{}^{t-1}ID_{1}\ldots ID_{1}ID_{3}{}^{t-1}-ID_{3}ID_{1}{}^{t-1}ID_{3}{}^{t}-ID_{1}{}^{t}\\ \ldots \\ ID_{t}{}^{t}-ID_{t-1}{}^{t}ID_{t}{}^{t-1}ID_{t-1}-ID_{t-1}{}^{t-1}ID_{t}\ldots ID_{t}ID_{t-1}{}^{t-1}-ID_{t-1}ID_{t}{}^{t-1}ID_{t-1}{}^{t}-ID_{t}{}^{t} \end{array}]\left[\begin{array}{l} a_{1}\\ a_{2}\\ \ldots \\ a_{t} \end{array}\right]$$

Since $ID_{1},ID_{2},\ldots ,ID_{t}$ are known identity values, the left matrix of Equation (62) is actually a coefficients matrix and Equation (63) is a set of t-order equations about $a_{1},a_{2},\ldots ,a_{t}$, it is easy to calculate the values of $a_{1},a_{2},\ldots ,a_{t}$ and make out the symmetric polynomial f(x,y), which shows that enemy can obtain the session key of the captured node and steal the information. Similarly, the whole network will be broken if the enemy captures enough nodes. The proof is thus finished. □

Unlike the Corollary 3 about the E-G scheme in which is hard to resist t-collusion attacks, in LCKMS-QPLIP, an independent and unique asymmetric n-ary quadratic private function g(x) has been preset for each node during the initialization stage. Firstly, it breaks through the conventional method of generating session key and uses multivariate asymmetric polynomials to expand the field of building session key based on polynomial pre-distribution scheme. Secondly, it changes the idea of E-G scheme and q-composite scheme of storing multiple polynomials to improve the key sharing rate. Thirdly, each node only stores a unique quadratic polynomial and generates an independent session key with each neighbor node, which can save the storage space and computing cost.

According to the definition of quadratic polynomial, the key problem of solving the n-ary quadratic polynomial is to obtain all elements of matrix A. While considering the symmetry of matrix A, it is needed to solve $\frac{n\left(n+1\right)}{2}$ elements including the diagonal elements and the elements of above or below the diagonal of matrix A. According to Corollary 3, E-G uses the symmetry of binary t-th-order symmetric polynomials to build the session key, and it can be broken as long as t related neighbors is obtained by enemy.

For LCKMS-QPLIP, firstly, each node is preset with an asymmetric n-ary quadratic polynomial whose characteristic of multivariate asymmetric polynomial enhances the complexity and irregularity of the algorithm, and the external attackers cannot set up the polynomial groups like Equation (61) to break the matrix by obtaining the nodes’ neighbor lists. Secondly, because each quadratic polynomial is independent and unique, it is not useful to capture other nodes. Thirdly, based on the above analysis of matrix A, the attacker needs to solve $\frac{n\left(n+1\right)}{2}$ elements to break the quadratic polynomial, and it is obvious that the difficulty of breaking will increase greatly as long as the dimension n of the quadratic changes slightly, which is far greater than the security of E-G.

**Figure 4:**
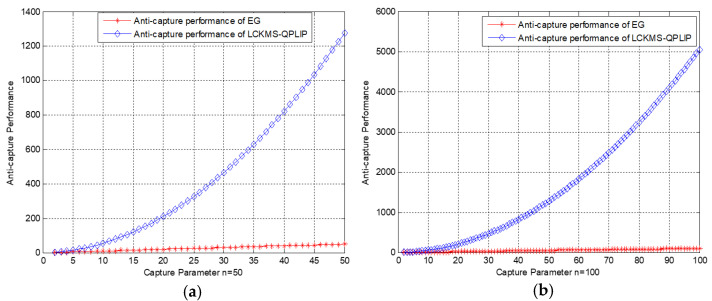
Anti-capture analysis n = 50 (**a**) & 100 (**b**).

In order to illustrate the difficulty and intuitiveness of breaking LCKMS-QPLIP, with the help of the idea of breaking E-G (the session key built by symmetric function is difficult to resist t-collusion attack), it is assumed that the parameter n is the order of binary symmetric polynomial in E-G scheme and that n also represents the number of quadratic polynomial’s variables in LCKMS-QPLIP. From the above analysis, it is known that the E-G scheme is difficult to resist the n-collusion attack. While for LCKMS-QPLIP, it is needed to break the private quadratic polynomial $g\left(x_{1},x_{2},\ldots ,x_{n}\right)$ which means that at least $\frac{n\left(n+1\right)}{2}$ parameters need to be obtained from matrix A (it is the minimum difficulty of breaking function based on the assumption that $g\left(x_{1},x_{2},\ldots ,x_{n}\right)$ is a symmetric polynomial). Figure 4 shows the comparison of anti-capture between the two schemes based on parameter n.

It can be seen in Figure 4 that with the increase of captured parameter n, the anti-capture ability of E-G scheme is linearly proportional change and it is possible to threaten the network as long as the enemy captures nodes of the same proportion. In contrast, LCKMS-QPLIP in this paper does not have this problem, since the anti-capture property changes exponentially, the larger the parameter n is, the more obvious the advantage is. The network is absolutely safe as long as it can guarantee $\frac{n\left(n+1\right)}{2}>N$, where N is the network size, because the number of network nodes is not enough to support the enemy to break any quadratic proportional.

#### 5.3.2. Anti-Capture Analysis of Broadcast Authentication Key

According to the above scheme of broadcast authentication key, the anti-capture property of broadcast authentication key is to ensure that the single captured node will not affect the security of broadcast authentication scheme, and the key factor is security of the shared function f(x). Once f(x) is leaked, it will affect the security of authentication, which illustrates that the pattern of f(x) is very important.

In order to detect the security or anti-capture property of f(x), it is assumed that f(x) is also a quadratic polynomial and also a continuous and integrable function about one variable $x_{i}$ on [−π,π], and the specific sharing function is $f\left(x_{i}\right)$.

According to security analysis in last step, it is needed to break the symmetric matrix A for breaking $f\left(x_{i}\right)$. While for breaking A, it is needed to obtain $\frac{n\left(n+1\right)}{2}$ elements including the diagonal elements and the elements of above or below the diagonal of matrix A, which means that $f\left(x_{i}\right)$ is absolutely safe as long as $\frac{n\left(n+1\right)}{2}>N$, where N is the network size.

#### 5.3.3. Anti-Capture Analysis of Group Key, Network Key and Personal Key

It is known from LCKMS-QPLIP that the security of group key, network key and personal key are consistent, and there are two main factors that affect the security of these three keys.

The one is the base station. Since these three keys are generated randomly by BS according to the former proposed schemes and it is hard to capture a BS, the source of key generation is quite safe.

The other one is the key information m(i). It is known that all nodes in the cluster rely on m(i) to obtain group key $K_{CH_{j}}$ and personal key $K_{S_{ij},BS}$, and the cluster head also obtains network key $K_{N}$ through m(i).

While according to the building process of these three keys, m(i) is the key to obtain these keys, and m(i) is encrypted by $K_{S_{ij},CH_{j}}$ and $K_{BS,CH_{j}}$, which means that it is needed to obtain $K_{S_{ij},CH_{j}}$ and $K_{BS,CH_{j}}$ for obtaining  m(i).

It is indicated from above equivalent security relationship that the security of m(i) is equivalent to the security of group key, network key and personal key, and the security of m(i) is also equivalent to the security of session key, which means the anti-capture property of group key, network key and personal key is equivalent to the anti-capture property of session key. It is known from above analysis that and the anti-capture property of session key can be reflected by the relation $N<\frac{n\left(n+1\right)}{2}$.

### 5.4. Efficiency Analysis

The efficiency of the proposed scheme LCKMS-QPLIP includes delay, storage and computation cost. The first author of this paper has discussed some efficiency of the scheme in the proposed literature [42,44].

(1) Authentication delay cost in [42]

It is indicated from Figure 5 to Figure 8 in [42] that the authentication delay of the proposed two protocols are all increased with the time changes, but the authentication delay of µTESLA are increased much faster with the authentication calculation increasing, while the authentication delay of MBAP included in LCKMS-QPLIP is changed stably.

(2) Storage cost in [44]

The storage cost of the proposed three schemes (LKH, EBS, AGKMS) for common sensor nodes are shown in Figure 5 of [44], and it is indicated that AGKMS included in LCKMS-QPLIP is much better than LKH and EBS in storage cost.

(3) Computation cost in [44]

The computation cost of the proposed three schemes (LKH, EBS, AGKMS) is shown in Figure 6 of [44], and it is indicated that the computation cost of AGKMS included in LCKMS-QPLIP in the situation of existing one captured node is much better than LKH and EBS.

Specially, compared with LKH and EBS, the computation cost for new node joining in LKH and EBS is very small because of the management by GC. Though the computation cost for new node joining in AGKMS a little larger than LKH and EBS, AGKMS scheme does not affect the structure of the network for new nodes and has a good scalability, and AGKMS can avoid the collusion problem and keep more security so, the AGKMS included in LCKMS-QPLIP in this paper has a good computation cost.

### 5.5. Network Robustness Analysis

In LCKMS-QPLIP, each network node has been preset a sharing function f(x) and a private function g(x) at the network initialization stage and sends the neighbor list information encrypted by the time-based authentication key to BS, which indicates that there is no plaintext information transmitted when the information begins to interact each other. For external attackers, they are unable to participate in any network information interaction because of the lack of f(x) and g(x). Since each session key is calculated by the key information of each two neighbor nodes, the attacker cannot obtain the session key directly from a single node without knowing the key calculation protocol. According to the above analysis of equivalent security, the security of other keys can be guaranteed if the security of the session key is ensured.

(1) Anti-collusion attack capability

Since the private quadratic polynomials g(x) are multivariate asymmetric polynomials, they are impossible to be obtained by attackers based on the collusion attack same as E-G scheme and q-composite scheme. Therefore, LCKMS-QPLIP can resist collusion attacks.

(2) Anti-flooding attack capability

An attacker can launch an attack flooding attack that the attacker can fake various identities and reply many forged messages to node a, and node a needs to authenticate these identities after receiving these messages. Each authentication requires a certain amount of computation, so that the attackers can send a lot of messages to consume the energy of a.

While LCKMS-QPLIP can resist such attacks, and the attackers cannot participate in any information interaction without the sharing function f(x) and private function g(x).

(3) Authentication analysis

In LCKMS-QPLIP, neighbor nodes can exchange their key information, calculate each other’s eigenvalues and eigenvectors, and judge the correctness of orthogonal matrix and symmetric matrix to complete the identity authentication. While these random key pre-distribution schemes such as E-G and q-composite can’t support the identity authentication of neighbor nodes, and it is vulnerable to disclose the keys when the nodes are captured by attackers.

(4) Scalability analysis

In the initialization stage of LCKMS-QPLIP, network nodes only need to be preset ID, f(x) and g(x). When a new node a is added, BS will preset ID, f(x), g(x) and current group key. The new node a broadcasts its own ID encrypted by the group key and establishes the neighbor list after obtaining all neighbor nodes’ ID. The new node a broadcasts its own key information encrypted by the group key and all neighbor nodes also send their own key information to node a after receiving the key information, and then building the session key between new neighbors based on above session key scheme.

In the whole process, the neighbor nodes only need to add the session key with the new node, and the irrelevant nodes have not changed, which means that the addition of new node does not affect any communication structure of the network. So, the LCKMS-QPLIP has strong scalability.

In addition, LCKMS-QPLIP is applicable to almost all symmetric cryptosystems and lightweight crypto-algorithms, and it does not rely on the additional auxiliary equipment and can be applied to various scales of wireless sensor networks.

## 6. Conclusions

The proposed layer-cluster key management scheme LCKMS-QPLIP in this paper has five important parts, it can guarantee the identity of network nodes through forward authentication and reverse authentication, and session key, group key and network key can guarantee the security and efficiency of network, and personal key can guarantee the privacy of network. These five keys complement each other, which not only ensures the independence of the keys’ management and avoids the problem of single point failure, but also enables WSN to make an efficient key management in a reasonable network structure.

## Figures and Tables

**Table 1:** Explanation of symbols.

Symbols	Explanation
BS/KDS	base station/key distribution center
$S_{ij}$	node i of cluster j
$CH_{j}$	cluster head j
h(x)	hash function
m(i)	key information of node i
$ID_{i}$	identity symbol of node i
f(x)	sharing function
g(x)	private function
$K_{a,b}$	session key between node a and node b
$K_{CH_{i},BS}$	session key between cluster i and BS
$K_{i,CH_{j}}$	session key between node i and cluster head j
$K_{j}$	group key of cluster j
$K_{S_{ij},BS}$	personal key of node i
$K_{N}$	network key
L(i)	broadcast authentication information
